# Analysis of Phosphorylation of the Receptor-Like Protein Kinase HAESA during Arabidopsis Floral Abscission

**DOI:** 10.1371/journal.pone.0147203

**Published:** 2016-01-19

**Authors:** Isaiah Taylor, Ying Wang, Kati Seitz, John Baer, Stefan Bennewitz, Brian P. Mooney, John C. Walker

**Affiliations:** 1 Division of Biological Science, University of Missouri, Columbia, Missouri, United States of America; 2 Interdisciplinary Plant Group, University of Missouri, Columbia, Missouri, United States of America; 3 Charles W. Gehrke Proteomics Center and Division of Biochemistry, Bond Life Sciences Center, University of Missouri, Columbia, Missouri, United States of America; University of Padova, ITALY

## Abstract

Receptor-like protein kinases (RLKs) are the largest family of plant transmembrane signaling proteins. Here we present functional analysis of HAESA, an RLK that regulates floral organ abscission in Arabidopsis. Through *in vitro* and *in vivo* analysis of HAE phosphorylation, we provide evidence that a conserved phosphorylation site on a region of the HAE protein kinase domain known as the *activation segment* positively regulates HAE activity. Additional analysis has identified another putative activation segment phosphorylation site common to multiple RLKs that potentially modulates HAE activity. Comparative analysis suggests that phosphorylation of this second activation segment residue is an RLK specific adaptation that may regulate protein kinase activity and substrate specificity. A growing number of RLKs have been shown to exhibit biologically relevant dual specificity toward serine/threonine and tyrosine residues, but the mechanisms underlying dual specificity of RLKs are not well understood. We show that a phospho-mimetic mutant of both HAE activation segment residues exhibits enhanced tyrosine auto-phosphorylation *in vitro*, indicating phosphorylation of this residue may contribute to dual specificity of HAE. These results add to an emerging framework for understanding the mechanisms and evolution of regulation of RLK activity and substrate specificity.

## Introduction

Receptor-like protein kinases (RLKs) are a highly expanded family of plant specific cell-surface receptor protein kinases. They constitute the largest group of transmembrane proteins encoded by plant genomes [1,2]. RLKs perceive a diverse set of stimuli, including steroid and peptide hormones, microbe-associated molecular patterns, and other exogenous and endogenous signals [Reviewed in [3–5]]. The RLK HAESA (HAE), along with the paralogous HAESA-LIKE 2 (HSL2), is required for post-pollination abscission of floral organs in Arabidopsis. During floral organ abscission, the outer three whorls of floral organs are shed from the growing silique in a developmentally programmed manner. A double mutant of *hae* and *hsl2* retains floral organs for the life of the plant and is therefore termed *abscission deficient* [6,7]. Genetic and biochemical analyses suggest HAE/HSL2 are activated by a secreted peptide derived from the *INFLORESCENCE DEFICIENT IN ABSCISSION (IDA)* gene, leading to activation of a MAP kinase cascade and subsequent activation of a transcriptional program leading to cell wall hydrolysis at the base of the abscising organs in a region of cells called the *abscission zone* [6,8–10].

HAE belongs to a large clade of RLKs characterized by possession of an extracellular leucine-rich repeat (LRR) domain, and is therefore termed an *LRR-RLK*. A number of studies have provided evidence for a suite of commonly occurring activation mechanism of many LRR-RLKs involving a combination of homo-oligomerization and protein kinase domain auto-phosphorylation, ligand induced hetero-association with co-receptors, and subsequent receptor/co-receptor trans-activation [11–24].Prior work with HAE has shown that recombinant HAE protein kinase domain is capable of auto-phosphorylating *in vitro* [25,26]. Analysis of HAE immuno-purified from abscission zones has further shown that natively expressed HAE is capable of incorporating radiolabelled ATP in an *in vitro* protein kinase assay [27]. Recent work utilizing a BiFC assay has shown that HAE can auto-associate when expressed in Arabidopsis mesophyll protoplasts [28]. These results together provide evidence that HAE functions as an auto-associating/auto-phosphorylating LRR-RLK.

To date, there has been no additional functional characterization regarding the impact of site specific phosphorylation on the activity of the HAE protein kinase domain. To understand the mechanism by which phosphorylation might regulate the biological activity of HAE, we developed a system to identify *in vitro* auto-phosphorylation sites on HAE and to test their importance for HAE activity by mutational analysis coupled with *in vitro* auto-phosphorylation and *in vivo* complementation assays. Our results implicate 2 serine residues (S856 and S861), located on the HAE activation segment, as critical regulators of HAE function. The activation segment is a conserved region of protein kinases that commonly harbors activating phosphorylation sites and contains elements involved in substrate binding [29–31]. Biochemical and comparative analyses suggests phosphorylation of S861 and the homologous residue in related protein kinases may be an RLK specific modulator of protein kinase activity. Multiple RLKs have recently been shown to possess dual specificity toward both serine/threonine and tyrosine residues [32–34]. We show that a double phospho-mimetic substitution mutant at the S856/S861 residues exhibits enhanced tyrosine auto-phosphorylation *in vitro*, suggesting differential phosphorylation of HAE at this non-canonical site in the activation segment may regulate substrate specificity.

This work provides novel genetic and biochemical findings pertinent to a general model of activation of RLKs and presents several testable hypotheses regarding regulation of activity of this large and important plant protein kinase family.

## Results

### A protein kinase inactive mutant of HAE is unable to complement the abscission deficient phenotype of a *hae hsl2* mutant

To investigate the importance of protein kinase activity on the signaling capacity of HAE, we tested the ability of a HAE-YFP fusion protein expressed by the *HAE* promoter to complement the abscission deficient phenotype of the *hae-3 hsl2-3* double mutant [9]. In parallel we tested the complementation ability of a mutant form (*HAEpr*::*HAE-YFP K711E)* of the same construct with a substitution of a conserved catalytic lysine known to be critical for protein kinase activity [35]. This mutation has been previously shown to abolish HAE protein kinase activity *in vitro* [25]. The *hae-3 hsl2-3* mutant contains single amino acid substitutions in the extra-cellular LRR domains of both HAE and HSL2 causing C222Y and G360R substitutions, respectively [S1 Fig]. RNA-Sequence analysis of this mutant demonstrated a large reduction in transcript abundance of cell wall hydrolytic enzymes, indicating the abscission defect in *hae-3 hsl2-3* is largely the result of inadequate breakdown of the middle lamella between abscising organs and the base of the silique in the abscission zone [9].

**Fig 1:**
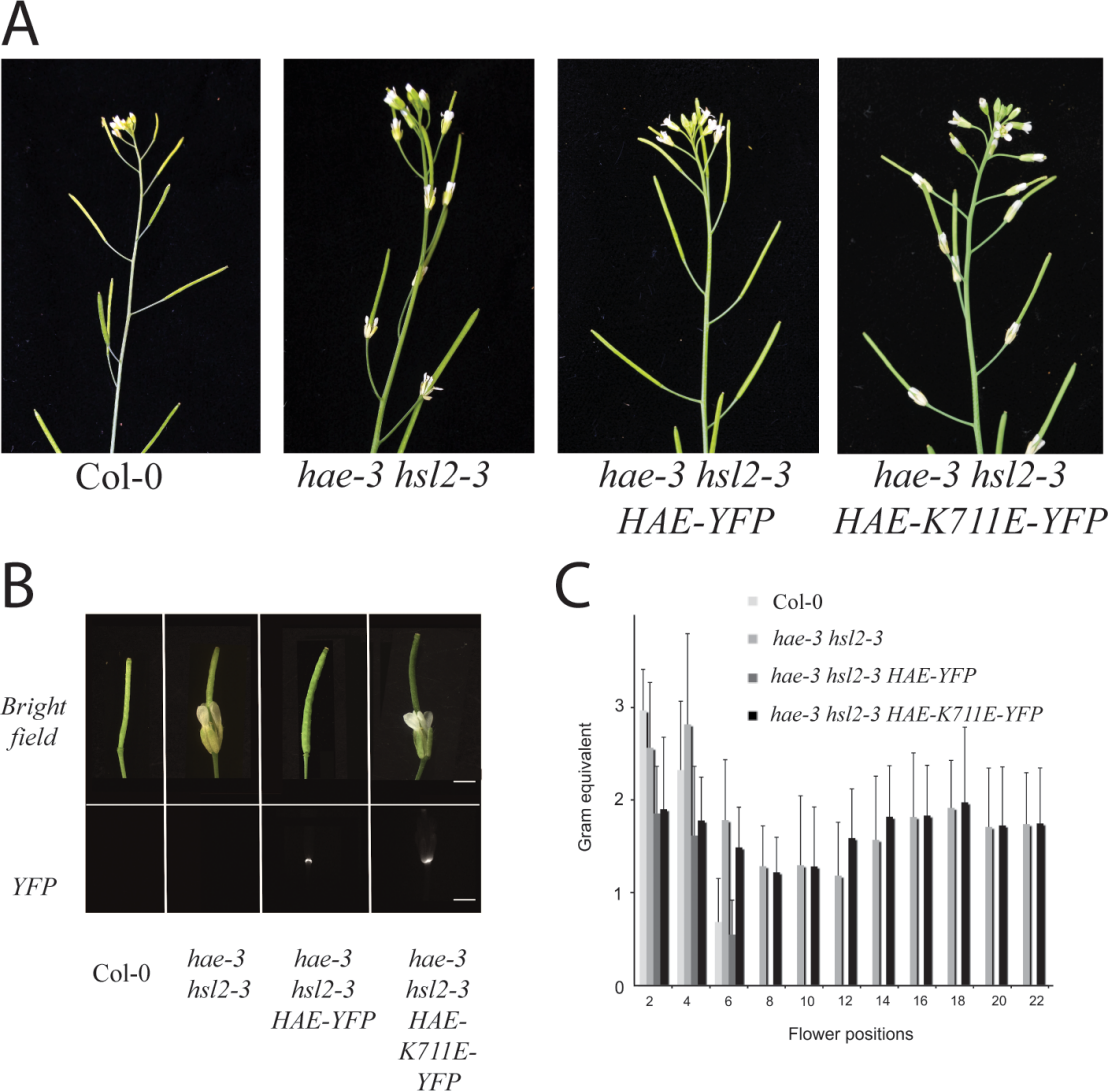
A protein kinase-inactive mutant of HAE is non-functional *in vivo*. A) Abscission phenotype of wildtype Col-0, *hae-3 hsl2-3*, *hae-3 hsl2-3 HAE-YFP*, and *hae-3 hsl2-3 HAE-YFP K711E*. B) HAE-YFP and HAE-YFP K711E accumulation in abscission zones. C) Breakstrength measurements comparing complementation ability of HAE-YFP to HAE-YFP-K711E.

Consistent with our hypothesis that protein kinase activity is required for HAE function, the *HAEpr*::*HAE-YFP* construct was able to efficiently rescue the abscission deficient phenotype of *hae-3 hsl2-3*, while the *HAEpr*::*HAE-YFP K711E* construct was completely unable to rescue the defect in any of the transgenic lines examined (n>30 for both constructs) [Fig 1A]. The inability of *HAEpr*::*HAE-K711E* to complement *hae-3 hsl2-3* does not appear to be an effect of alterations in protein level, as multiple abscission deficient T1 lines displayed strong YFP signal in the abscission zones [Fig 1B]. Quantitative analysis using a petal breakstrength meter to measure force required to remove non-abscised floral organs confirms that *HAEpr*::*HAE-YFP K711E* has no measurable ability to complement the *hae-3 hsl2-3* abscission deficient phenotype [Fig 1C]. These results suggest that protein kinase activity is required for biological function of HAE.

### MS/MS analysis of recombinant HAE intracellular domain identifies 9 *in vitro* auto-phosphorylation sites

**Fig 2:**
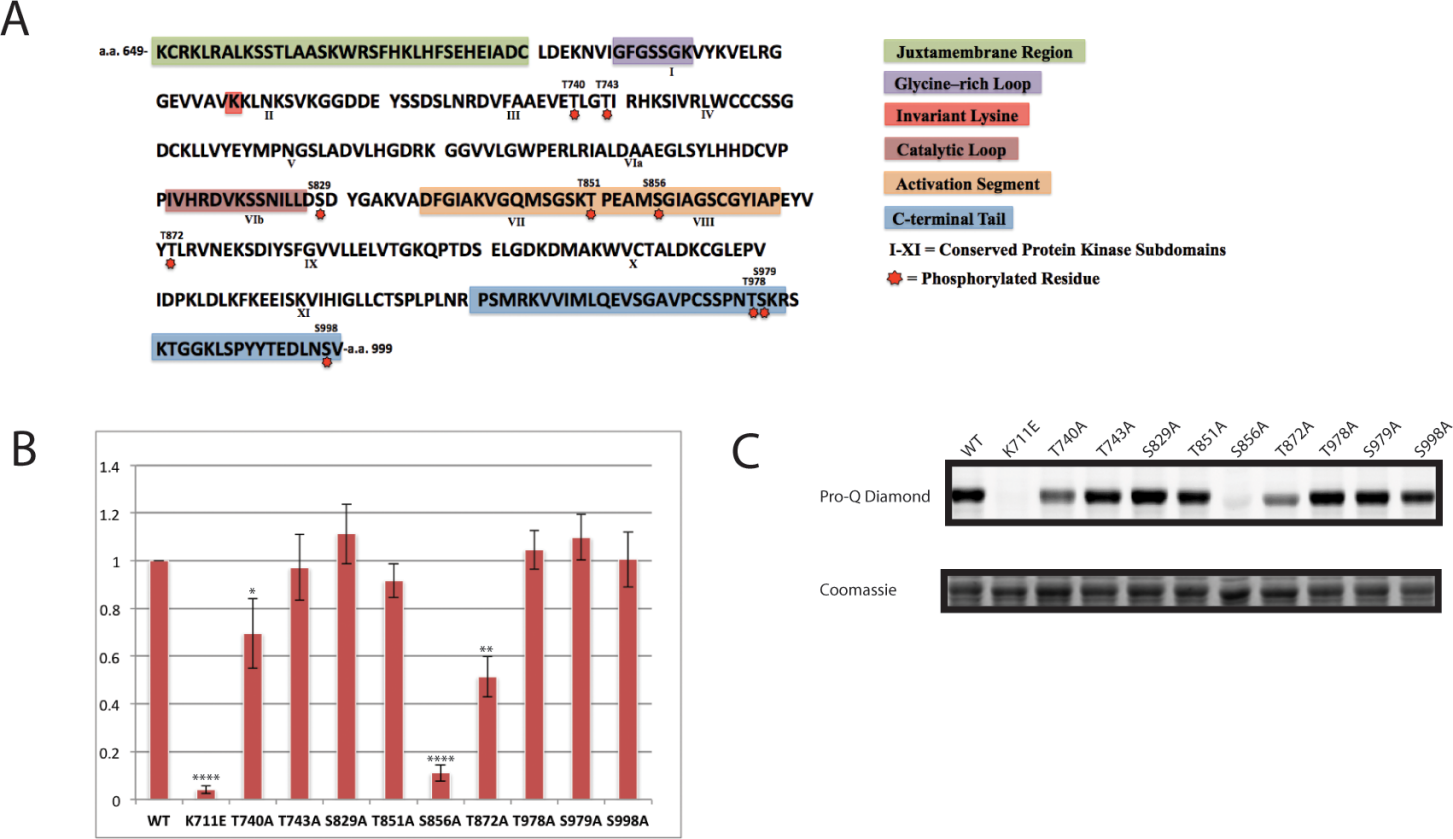
Functional analysis of HAE *in vitro* auto-phosphorylation sites. A) Identified HAE auto-phosphorylation sites annotated on the amino acid sequence of the HAE intracellular domain. Conserved features of protein kinases, as well as the HAE specific juxtamembrane region and C-terminal tail, are annotated for reference. B) Ratio of mutant:wildtype *in situ* auto-phosphorylation levels of recombinant MBP-HAE mutants. N = 4 biological replicates. Wildtype is normalized to 1. Error bars represent the standard deviation. Tests for statistical significance were carried out by a one sample t-test. C) Representative stains for phosphorylation and total protein by Pro-Q Diamond and coomassie blue, respectively.

Work focusing on a number of RLKS has demonstrated that recombinant protein kinase domains of many RLKs expressed in *E*. *coli* auto-phosphorylate to a very high level in bacterial cells prior to any purification [26,36–38]. Oh *et al*. have termed this phenomenon *in situ* auto-phosphorylation, to identify it as a special case of more general *in vitro* auto-phosphorylation of recombinant protein kinases, which may occur either prior to or after protein purification [37]. To identify phosphorylation sites important for HAE activity, we subjected *in situ* auto-phosphorylated GLUTATHIONE S-TRANSFERASE(GST)-tagged HAE intracellular protein kinase domain (previously shown to be an active protein kinase *in vitro* [25]) to MS/MS based phosphorylation site identification. Intact mass analysis of undigested protein showed there exists a population of different phospho-isoforms phosphorylated between 3 and 9 times [S2 Fig]. Peptide sequencing of Trypsin and Glu-C digested GST-HAE led to the unambiguous identification of 9 phosphorylation sites on the HAE protein kinase domain [Fig 2A]. These sites have been annotated on the HAE intracellular domain protein sequence. For reference, we have annotated a number of widely conserved subdomains of protein kinases identifiable from primary amino acid sequence [39,40]. MS/MS data are presented in S1 Table.

### *In vitro* and *in vivo* analysis supports S856 in the activation segment as a phosphorylation site that positively regulates HAE protein kinase activity

To attempt to understand the functional impact on HAE activity of phosphorylation at the identified sites, we performed alanine substitution mutagenesis individually at all 9 sites on a plasmid encoding the HAE intracellular kinase domain tagged with an N-terminal Maltose Binding Protein(MBP) affinity/solubility tag. This construct has been previously shown to encode an active protein kinase *in vitro* [25]. Next we measured the relative auto-phosphorylation compared to wildtype in 4 biological replicates using a previously described purification-free *in situ* protein kinase assay [26]. This assay involves expressing recombinant HAE intracellular domain in *E*. *coli*, followed by SDS-PAGE of the boiled cell lysate, and sequential staining with the phospho-amino acid stain Pro-Q Diamond and a total protein stain. The background-adjusted Pro-Q Diamond/total protein ratio yields a quantitative measure of protein auto-phosphorylation. This assay is rapid and consistent, though it should be noted reductions in observed auto-phosphorylation for a given mutant can be attributable to either reduced protein kinase activity, or alternatively to simple elimination of a non-essential phosphorylation site that nonetheless contributes to Pro-Q Diamond staining. Despite this caveat, dramatic changes in auto-phosphorylation are indicative of major alterations in protein kinase function.

**Fig 3:**
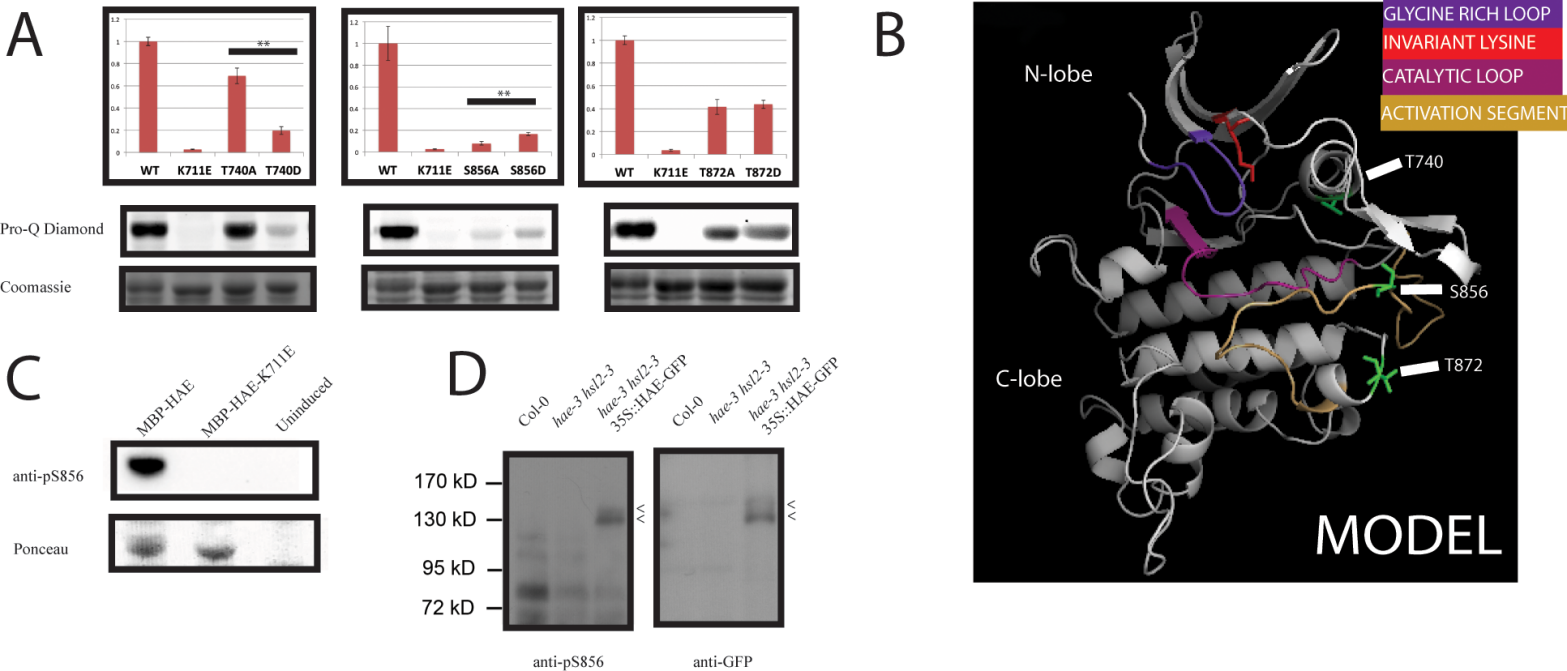
Evidence that S856 is an *in vivo* phosphorylation site that positively regulates HAE protein kinase activity. A) Quantified *in situ* auto-phosphorylation levels for alanine and aspartate substitution mutants at 3 in vitro phosphorylation sites averaged over 3 biological replicates. ** = p-value < .01. Error bars represent standard deviation. Tests for statistical significance were carried out by performing a 2 sample t-test. B) Model of HAE protein kinase with selected phosphorylation sites annotated in green. C) Detection of active MBP-HAE by anti-pS856 anti-body in whole cell lysate from MBP-HAE expressing *E*. *coli*. D) Specific detection of a protein migrating at the expected size (~130 Kda) with the anti-pS856 and anti-GFP antibodies in protein isolated from Arabidopsis flowers overexpressing HAE-GFP by the 35S promoter. Arrows mark potential phospho-isoforms.

This experiment identified 3 mutants, T740A, S856A, and T872A, with significantly lower levels of auto-phosphorylation than the wildtype control [Fig 2A and 2B], and none with higher levels of auto-phosphorylation. S856 is a notable site because it aligns to a conserved portion of the activation segment occupied by the classical, primary activating phosphorylation site in a number of protein kinases [29,40]. T740 resides in the subdomain III alpha helix, while T872 resides C-terminal to the activation segment adjacent to a predicted short alpha helix. We created a homology model of HAE based on a recently solved BRI1 protein kinase domain crystal structure with these sites annotated to provide a rough map of their spatial location on the HAE protein kinase domain [Fig 3B].

To corroborate the possible roles that phosphorylation at these three residues plays in regulating activity of HAE *in vitro*, we substituted each residue with the negatively charged amino acid residue aspartate to attempt to mimic constitutive phosphorylation. The results in Fig 3A show that the T740D mutant has a further reduction in auto-phosphorylation compared to T740A, suggesting that this threonine is important for protein kinase activity but making it difficult to determine what impact, if any, phosphorylation at this site has on HAE activity. T872D has indistinguishable levels of auto-phosphorylation from T872A, again making it difficult to infer what effect, if any, phosphorylation at this site may have on protein kinase activity. The S856D mutant, however, displays a modest but highly repeatable increase of ~2 fold over the corresponding S856A mutant. This suggests the S856D mutation weakly mimics the activated state of the HAE kinase, providing support for the hypothesis that S856 is an activating phosphorylation site.

To study the function of phosphorylation of S856, we raised a phospho-specific antibody against a peptide containing pS856. This antibody recognizes recombinant wildtype MBP-HAE, but not MBP-HAE-K711E [Fig 3C], providing strong evidence for the phospho-specificity of the antibody as well as the presence of pS856 on HAE *in vitro*.

We were unable to unambiguously detect HAE with this antibody in protein isolated from wildtype Arabidopsis flowers, reflecting a possible combination of low sensitivity of the antibody, low HAE abundance, and/or low stoichiometry of this precise phospho-isoform *in vivo*. We were, however, able to specifically detect a band of the expected size in protein isolated from flowers of a complemented *hae-3 hsl2-3* transgenic line overexpressing *HAE-GFP* driven by the *35S* promoter [Fig 3D] [S3 Fig], indicating that HAE can be phosphorylated *in vivo* on S856. The protein recognized by the anti-pS856 antibody appears to display a pattern of gel shift similar to that observed with an anti-GFP antibody used to probe a blot of the same samples, suggesting the antibodies are detecting the same protein. We hypothesize this gel shift is due to phosphorylation of the HAE-GFP protein, consistent with a model where the protein exists as multiply phosphorylated isoforms.

### The HAE-YFP S856A mutant displays a weakened ability to complement the *hae-3 hsl2-3* mutant

**Fig 4:**
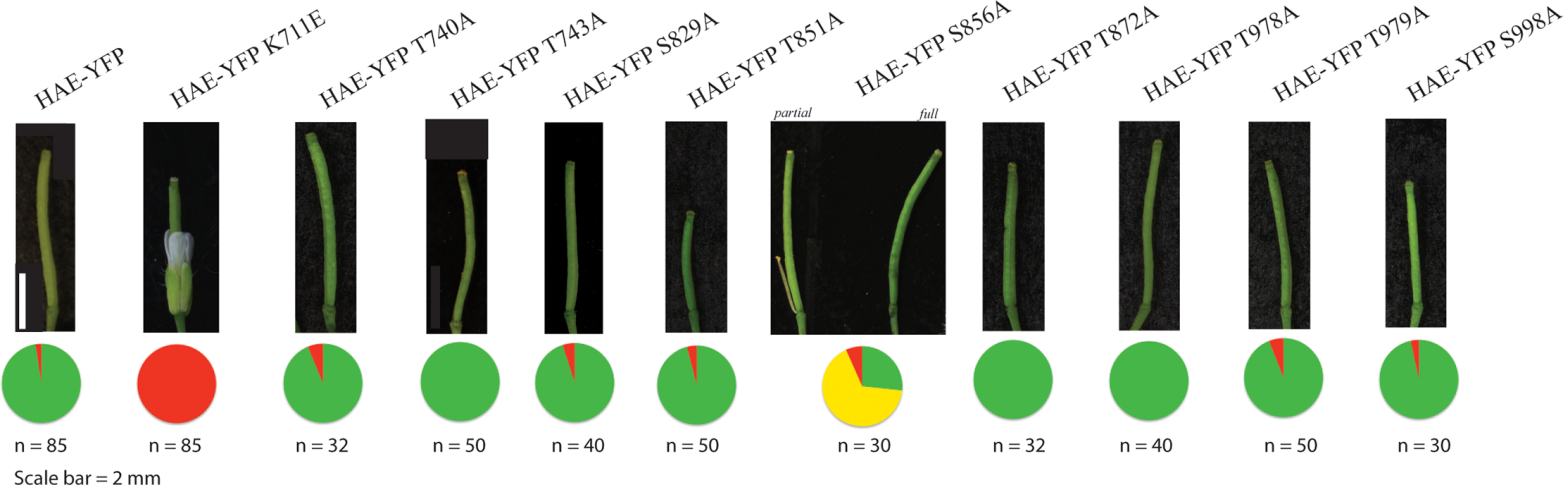
*In vivo* analysis of HAE in vitro auto-phosphorylation sites mutants. Complementation ability of mutants of HAE *in vitro* auto-phosphorylation sites. Green = full complementation, yellow = partial complementation, and red = non-complementation, N = number of T1 lines examined.

To test the role of the identified *in situ* phosphorylation sites in regulating HAE activity *in vivo*, we created alanine substitution mutations at each site in the *HAEpr*::*HAE-YFP* expression construct described above and tested their ability to complement *hae-3 hsl2-3*. As observed before, the *HAEpr*::*HAE-YFP* wildtype construct was able to complement at a high efficiency in the T1 (>97%, 83/85 independent T1 lines), while the K711E mutant was completely unable to complement (0%, 0/85) [Fig 4A]. Of the 8 mutants besides S856A, each displayed a similar ability as wildtype to complement at a rate >90%. This result suggests that none of these identified *in situ* phosphorylation sites is essential for HAE function.

Analysis of abscising HAE-YFP wildtype T1 lines demonstrated wide variability in YFP expression, from barely above our detection threshold to extremely high [Figure A in S4 Fig]. In contrast, examination of two non-abscising HAE-YFP T1 lines showed that neither possessed detectable YFP expression at all, indicating that at least a small amount of HAE protein is necessary and sufficient to rescue the abscission defect of *hae-3 hsl2-3* [Figure A in S4 Fig].

We hypothesized that the S856A mutant, like the K711E mutant, would be completely unable to complement *hae-3 hsl2-3*, based on the nearly total elimination of auto-phosphorylation of the S856A mutant *in vitro*. However, analysis of a population of T1 individuals expressing the *HAEpr*::*HAE-YFP S856A* mutant transgene was instead surprisingly composed of individuals that we could group into 3 categories: 27% (8/30) T1 plants exhibited complete abscission and were indistinguishable from wildtype. 67% (20/30) of T1 plants abscised the majority of their floral organs but retained a small number of non-abscised floral organs and were termed *partially rescued*. 6% (2/30) of T1 individuals did not abscise at all. Representative siliques for the partially and fully complemented T1 lines are displayed in Fig 4A. Figure B in S4 Fig contains representative inflorescence photos. These data suggests the S856A mutant possesses lower, but still substantial, biological activity.

During analysis of *HAE-YFP S856A* T1 plants, we also observed wide variation in HAE-YFP S856A expression [Figure C in S4 Fig]. Interestingly, and contrary to our hypothesis, the level of expression did not appear to be tightly correlated with the extent of abscission rescue. Multiple low YFP expressing lines displayed full abscission, and multiple high expressing T1 lines displayed partial abscission [Figure C in S4 Fig]. These data suggest that the S856A mutant is partially impaired in activity, but that there are other varying endogenous factors that control the extent of abscission in these lines in addition to HAE protein level. This may indicate that while HAE is essential for abscission, its abundance is not the limiting factor during activation of abscission signaling. Similar to the case of wildtype HAE-YFP, analysis of a completely non-abscising T1 line for the S856A mutant showed that it did not display any detectable YFP expression, suggesting that at least a low level of HAE is necessary for abscission to occur [Figure D in S4 Fig].

Because of the unexpected ability of the S856A mutant to function, we performed two experiments to rule out possible artifactual causes for S856A complementation. First, to test that the YFP tag was not somehow inducing hyperactivation of the HAE kinase, leading to recovery of activity of the S856A mutant, we truncated the YFP tag by inserting a stop codon into the linker connecting the HAE coding sequence to the YFP coding sequence of the *HAEpr*::*HAE-YFP S856A* entry vector. This construct was then expressed in *hae-3 hsl2-3*, where we found it complemented at a similar efficiency as the YFP fusion [Figure A in S5 Fig] ruling out an effect of the YFP tag. Second, a recent report has demonstrated interallelic effects in plants expressing multiple RLK mutant alleles [41]. To test whether we were observing allele dependent complementation of *hae-3 hsl2-3* by the *HAEpr*::*HAE-YFP S856A* transgene, we expressed the *HAEpr*::*HAE-YFP S856A* construct in a recently generated mutant *hae-5 hsl2-4* with mutations causing premature stop codons in the extracellular domains [41] [S1 Fig]. We found the *hae-5 hsl2-4* mutant was complemented at a similar rate as *hae-3 hsl2-3* [Figure B in S5 Fig], ruling out an allele specific effect of the *hae-3 hsl2-3* background.

Despite the partial complementation ability of the S856A mutant, it still exhibits a clear reduction in functionality compared to the wildtype transgene. We therefore hypothesized that the S856D mutant might complement at a higher efficiency, given its increased relative auto-phosphorylation *in vitro*. Contrary to this hypothesis, transformation of the *HAEpr*::*HAE-YFP S856D* transgene into *hae-3 hsl2-3* yielded a population of plants exhibiting a spectrum abscission phenotypes which we could not distinguish from that of a population expressing *HAEpr*::*HAE-YFP S856A* [Figure A in S6 Fig]. These plants displayed varying levels of HAE-YFP accumulation similar to those seen for *HAE-YFP S856A* T1 plants [Figure B in S6 Fig]. We hypothesize the S856D phosphomimic is insufficiently similar to pS856 to cause a detectable enhancement of activity *in vivo* over the S856A mutant.

### Mutational analysis *in vivo* and comparative analysis identifies S861 as a possible phosphorylation site with a role in regulating HAE activity

**Fig 5:**
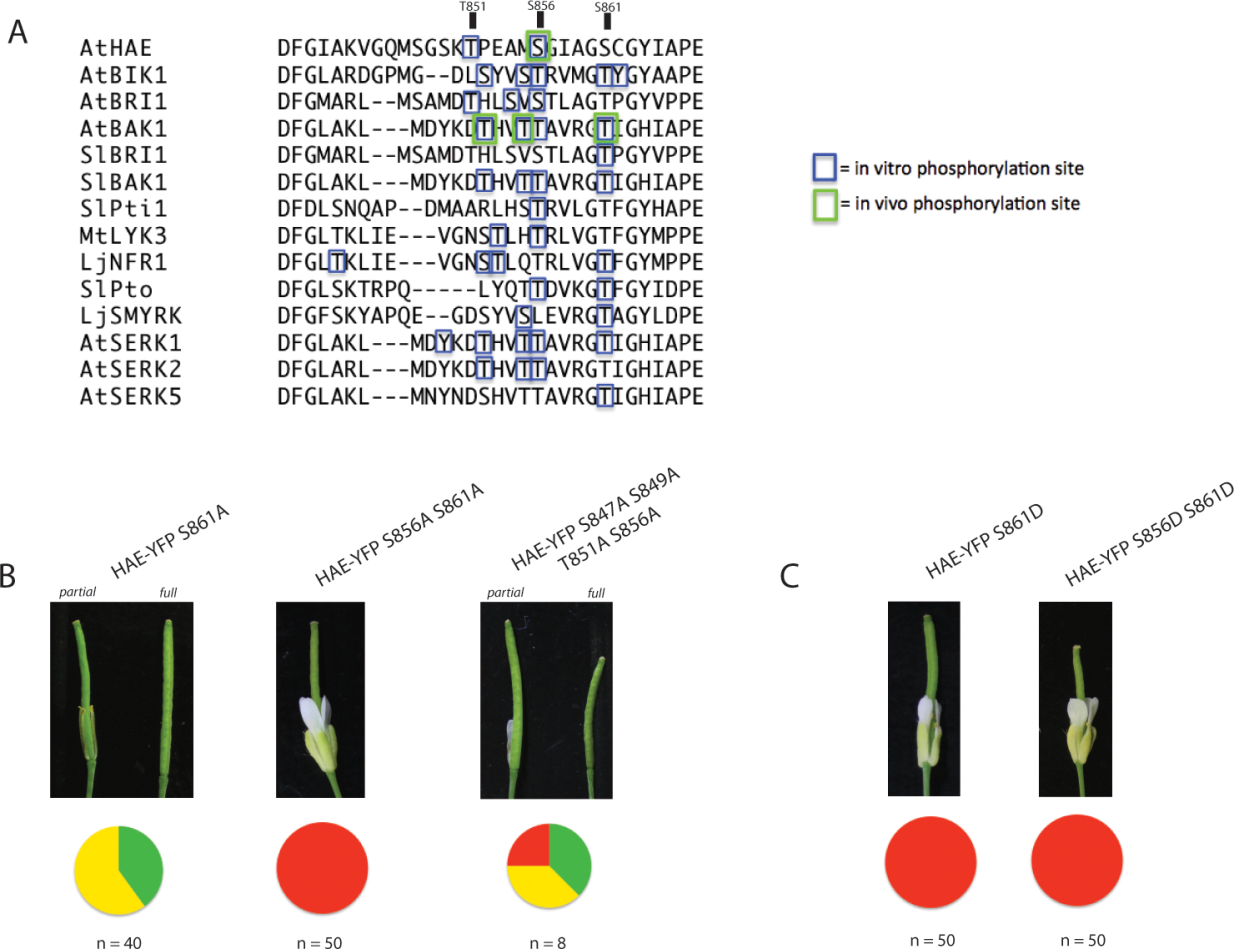
Evidence of coordinated regulation of HAE activity by the activation segment residues S856 and S861. A) Alignment of the activation segment of RLKs for which phosphorylation sites have been identified from the literature. Phosphorylation sites are indicated by boxes. B) Complementation ability of the HAE-YFP S861A single mutant, HAE-YFP S856A S861A double mutant, and HAE-YFP S847A S849A T851A S856A quadruple mutant. Green = full complementation, yellow = partial complementation, and red = non-complementation. C) Complementation ability of the HAE-YFP S861D single and HAE-YFP S856D S861D double mutants.

Some protein kinases contain multiple phosphorylation sites on the activation segment to regulate activity [40]. Based on the ability of the *HAE*-*S856A* mutant to partially function, we hypothesized that there may be one or more additional phosphorylation sites in the proximity of S856 that can support partial function even in the absence of pS856. One logical candidate is T851, based on its identification as an *in vitro* auto-phosphorylation site. To generate additional candidate sites, we performed a literature search for similar analyses of RLK family members in any plant species for which phosphorylation sites have been unambiguously mapped to the activation segment by mass spectrometry or crystallography and performed an alignment of the sequences [Fig 5A] [See materials and methods for search details] [22,31,34,42–53].

The resulting alignment indicates there are a large number of phosphorylation sites on the activation segment of many RLKs. Based on the results of our work and others, it appears RLKs in general display promiscuous auto-phosphorylation *in vitro* and that many of the identified sites may not be biologically relevant (at least 19 sites have been mapped to AtBIK1 [43], at least 12 to AtBRI1 [54], and 21 to AtHAE based on results above and those detailed below). As such, the identification of a phosphorylation site on any one RLK *in vitro* should be considered only weak evidence of functional importance.

From the alignment amino acids that align to HAE-S856 as well as S861 are both conserved S/T residues, and enriched in identified phosphorylation sites [Fig 5A]. A position in the vicinity of T851 also appears to commonly exhibit phosphorylation, though the sequence conservation is not as strict as the alignment to S856 and S861. Of particular importance may be that the homologous threonine to S861 has been identified as an *in vivo* phosphorylation site on AtBAK1 [31].

In addition Mitra *et al*. recently published a large dataset of RLK *in vitro* auto-phosphorylation sites containing 68 members of the LRR-RLK family [38]. Analysis of this dataset yields 41 protein kinases for which at least one activation segment phosphorylation site had been identified. There appears to be 3 regions exhibiting enrichment of phosphorylation sites: 59% (24/41) of protein kinases contain at least one phosphorylation site within a 4 amino acid residue region on the activation loop region aligning to HAE-P852-HAE-S856, 51% (21/41) contain a phosphorylation site aligning to HAE-A841, and 29% (12/41) contain a phosphorylation site aligning to HAE-S861. HAE itself was included in this analysis, and S856 was identified as an *in vitro* auto-phosphorylation site. It is unclear why a higher percentage of protein kinases from the literature exhibit phosphorylation at the site homologous to S861. It may be due to a skewed phylogenetic distribution of protein kinases contained within the previously published studies. Nonetheless, this work provides support for the hypothesis that the residue homologous to S861 is a common RLK auto-phosphorylation site.

To test the functional importance of various activation segment S/T residues in regulating HAE activity, we constructed an additional series of alanine substitution mutants for *in vivo* complementation experiments. This series includes the *HAEpr*::*HAE-YFP S861A* single and *HAEpr*::*HAE-YFP S856A S861A* double mutant. We hypothesized that these two sites may coordinately promote HAE activity and that these mutants would display impaired abscission. As a control, we also created the *HAEpr*::*HAE-YFP S847A S849A T851A S856A* quadruple mutant. This mutant contains alanine substitutions at all activation segment serine/threonine residue aside from S861. We predicted that, if our model of S856/S861 dual control of activation was correct, the quadruple mutant would resemble the single S856A mutant.

The results of this experiment are shown in [Fig 5B]. Consistent with our hypothesis, the *HAEpr*::*HAE-YFP S861A* single mutant displays reduced complementation efficiency comparable to the *HAEpr*::*HAE-YFP S856A* single mutant, where ~40% of T1 plants appear wildtype and the remaining plants display partial abscission [Fig 5B]. Similar to what was observed for the *HAE-YFP S856A* T1 lines, *HAE-YFP S861A* mutant T1 lines displayed varying levels of HAE-YFP accumulation [Figure A in S7 Fig]. In addition to a partial reduction in complementation ability of the S861A single mutant, the *HAEpr*::*HAE-YFP S856A S861A* double mutant displays a complete lack of floral abscission resembling the *HAEpr*::*HAE-YFP K711E* mutant [Fig 5B], demonstrating dependence of HAE activity on presence of either S856 or S861. The quadruple mutant, however, displayed full or partial abscission in a majority of T1 lines, similar to the S856A single mutant, specifically arguing against a function for T851 in regulating HAE activity. This result suggests that phosphorylation of S847, S849, and T851 are not required for HAE activation.

Based on the reduced ability of the S861A mutant to complement *hae-3 hsl2-3*, and the evidence that it may be a widespread RLK phosphorylation site, we hypothesized that the *S861D* single and *S856D S861D* double phospho-mimetic mutants might exhibit enhanced ability to complement the abscission defect of *hae-3 hsl2-3*. Contrary to this hypothesis, neither the single *HAEpr*::*HAE-YFP S861D* nor double *HAEpr*::*HAE-YFP S856D S861D* mutant was able to complement the abscission deficient phenotype to any detectable degree. Instead all T1 individuals resembled the *HAEpr*::*HAE-YFP K711E* mutant in their complete lack of abscission, despite displaying strong YFP signal [Fig 5C][Figure C in S7 Fig]. One possibility to explain these data is there are one or more critical molecular interactions with which the phospho-mimetic mutation at S861 interferes compared to genuine pS861. Alternatively, S861 may function not as an activating phosphorylation site, but rather it may promote HAE activity through interactions of the non-phosphorylated serine residue. Further, phosphorylation of this residue could even conceivably function as a negative regulator of HAE activity. These hypotheses are discussed below.

### MS/MS analysis of an MBP-HAE mutant identifies S861 as a low level *in vitro* phosphorylation site

Based on our comparative analysis and *in vivo* mutant analysis, we were interested in investigating the possibility that S861 is a HAE auto-phosphorylation site. It was not identified in our initial *in vitro* auto-phosphorylation MS/MS experiment. It is possible it is only phosphorylated *in vivo*, perhaps through the action of an activating co-receptor. It is also possible it is phosphorylated *in vitro* at a level below our original detection threshold. Reanalysis of our initial MS/MS data suggested that the tryptic peptide containing S861 was miscleaved at K850, possibly due to interference by phosphorylated T851, leading to presence of a larger tryptic fragment and complicating peptide sequencing [S1 Table]. Miscleavage preceding phosphorylated sites has been reported [55]. To eliminate possible miscleavage and to enhance sensitivity, we mapped phosphorylation sites of *in situ* phosphorylated MBP-HAE-T851A mutant to determine if we could detect phosphorylated S861. Because the HAE-T851A mutant exhibits no observable defect *in vitro* or *in* vivo, we consider it probable that it functions essentially as wildtype.

An initial analysis of MBP-HAE-T851A tryptic fragments indeed identified a peptide matching the size of one containing pS861 at a low level, but we were unable to confirm its sequence identity. A second replicate positively identified sequence of this fragment, confirming phosphorylation of S861 at a level substantially lower than pS856. We observed evidence of both single pS861, as well as double pS856 pS861. These results together suggest that only a small population of MBP-HAE-T851A molecules is phosphorylated on S861, compared to a much larger population phosphorylated only on S856. MS/MS data are presented in S1 Table.

A notable observation is that the MBP-HAE-T851A mutant protein analyzed in our first replicate exhibited between 0–5 phosphorylation sites by intact mass analysis [S2 Fig], which is fewer than the 3–9 observed on GST-HAE. The second replicate of MBP-HAE-T851A was allowed to auto-phosphorylate for 12 hours during induction, rather than 5.5 for the first replicate, and exhibits roughly 20% higher auto-phosphorylation than the first MBP-HAE-T851A replicate as measured by Pro-Q Diamond [S2 Fig]. We speculate GST-HAE exhibits enhanced auto-phosphorylation compared with MBP-HAE due to the dimerization ability of GST, leading to enhanced effective concentration of HAE protein kinase domains.

**Fig 6:**
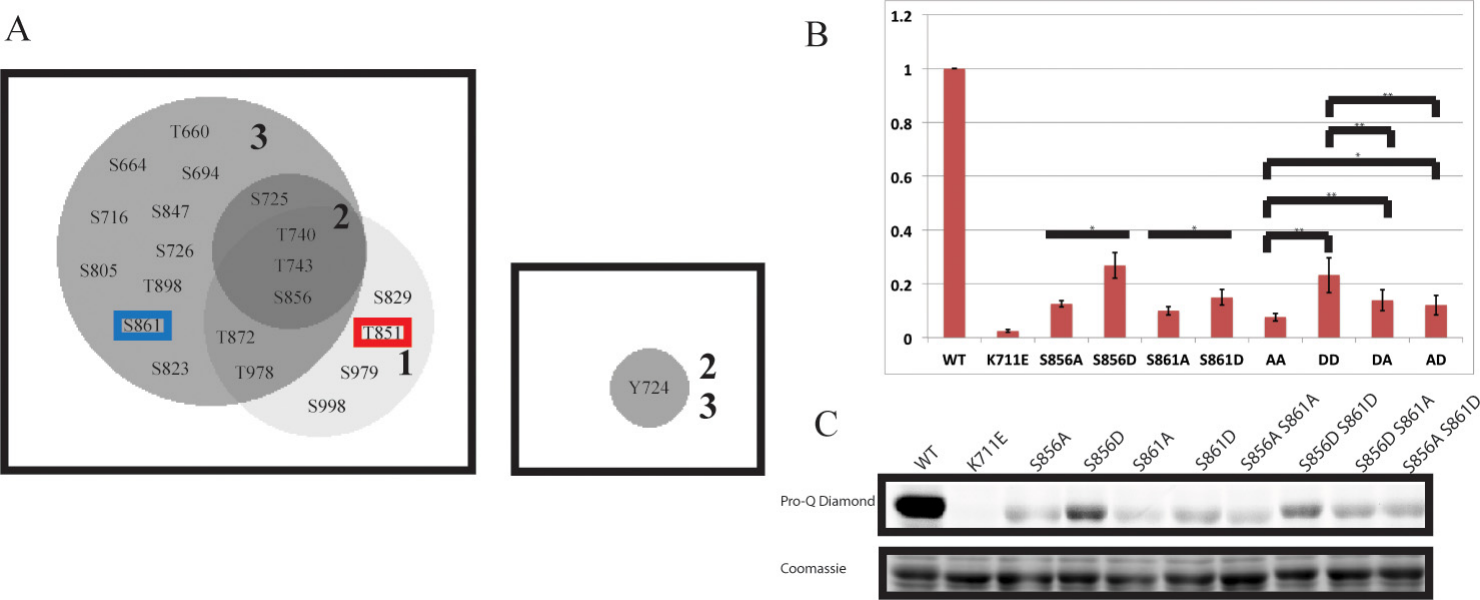
MS/MS analysis identifies S861 as a low level phosphorylation site. A) Venn diagram of phosphorylation sites identified in 3 replicates of MS/MS analysis *of in situ* auto-phosphorylated HAE. T851 is marked by a red box to indicate it was mutated in the protein analyzed in the 2nd and 3rd replicate, precluding its identification as a phosphorylation site. S861 is marked in blue to indicate the 2nd and 3rd replicates were conducted in order to identify this site. B) *In situ* auto-phosphorylation levels of activation segment mutants. N = 4 biological replicates. Error bars represent standard deviation. Statistical tests between the single mutants were carried out by a 2 sample t-test. Statistical tests to compare the 4 activation segment double mutants was performed by ANOVA with Tukey-Kramer correction for multiple comparisons. C) Representative stains for phosphorylation and total protein by Pro-Q Diamond and coomassie blue, respectively.

In addition to S861 and sites identified in our first replicate, we identified 9 more serine/threonine phosphorylation sites, as well as a single tyrosine, Y724. The sites identified in each of the 3 rounds of MS/MS analysis are displayed in a Venn diagram in Fig 6A. The abundance and variation of phosphorylation sites detected underscore the promiscuity of HAE *in vitro*, and advises strong caution in interpretation of these results in isolation. It is notable that there are only 3 sites identified in all 3 replicates, T740, T743, and S856. This suggests these may be relatively high occupancy *in vitro* auto-phosphorylation sites.

### Alanine substitution and phospho-mimetic analysis *in vitro* support the role of phosphorylation of S856 and S861 in positively regulating HAE activity *in vitro*

Because the MBP-HAE-T851A MS/MS experiment was essentially biased toward identifying phosphorylated S861, we view this as only weak evidence of the functional role of pS861. To test whether S861 is important for HAE activity *in vitro*, we created a series of single and double alanine and aspartate substitution mutant versions of MBP-HAE at positions S856 and S861 and performed similar *in situ* auto-phosphorylation analysis as before. Similar to what was observed for the S856A mutant, the S861A mutant has dramatically reduced *in vitro* auto-phosphorylation levels [Fig 6B and 6C]. There is a consistent and statistically significant ~50% increase in auto-phosphorylation in the MBP-HAE-S861D mutant compared to MBP-HAE-S861A. We also observed a small but statistically significant increase in auto-phosphorylation of the MBP-HAE-S856D S861A and MBP-HAE-S856A S861D double mutants over the MBP-HAE-S856A S861A double mutant, and a similar increase of the MBP-HAE-S856D S861D double over the MBP-HAE-S856D S861A and MBP-HAE-S856A S861D double mutants [Fig 6B and 6C]. In other words, these data show that aspartate substitutions increase MBP-HAE auto-phosphorylation over the corresponding alanine substitutions in this assay in all cases, and they are consistent with a role of both phosphorylated S856 and S861 in positively regulating HAE activity. Statistical comparisons were only carried out between mutants of the equivalent number of residues to control for reductions in observed auto-phosphorylation that may be attributable to simple elimination of a phosphorylation site, rather than alterations in protein kinase activity.

### The P + 1 loop of receptor-like protein kinases exhibit evidence of functional divergence from other eukaryotic protein kinases

A recently published crystal structure of the auto-phosphorylated protein kinase domain of the LRR-RLK BAK1 in an apparently active confirmation exhibits phosphorylation of T450 and T455 [44]. BAK1-T450 aligns to HAE-S856, and BAK1-T455 aligns to HAE-S861. This is consistent with a model of RLK activation where both of these conserved activation segment residues are phosphorylated to positively regulate activity.

**Fig 7:**
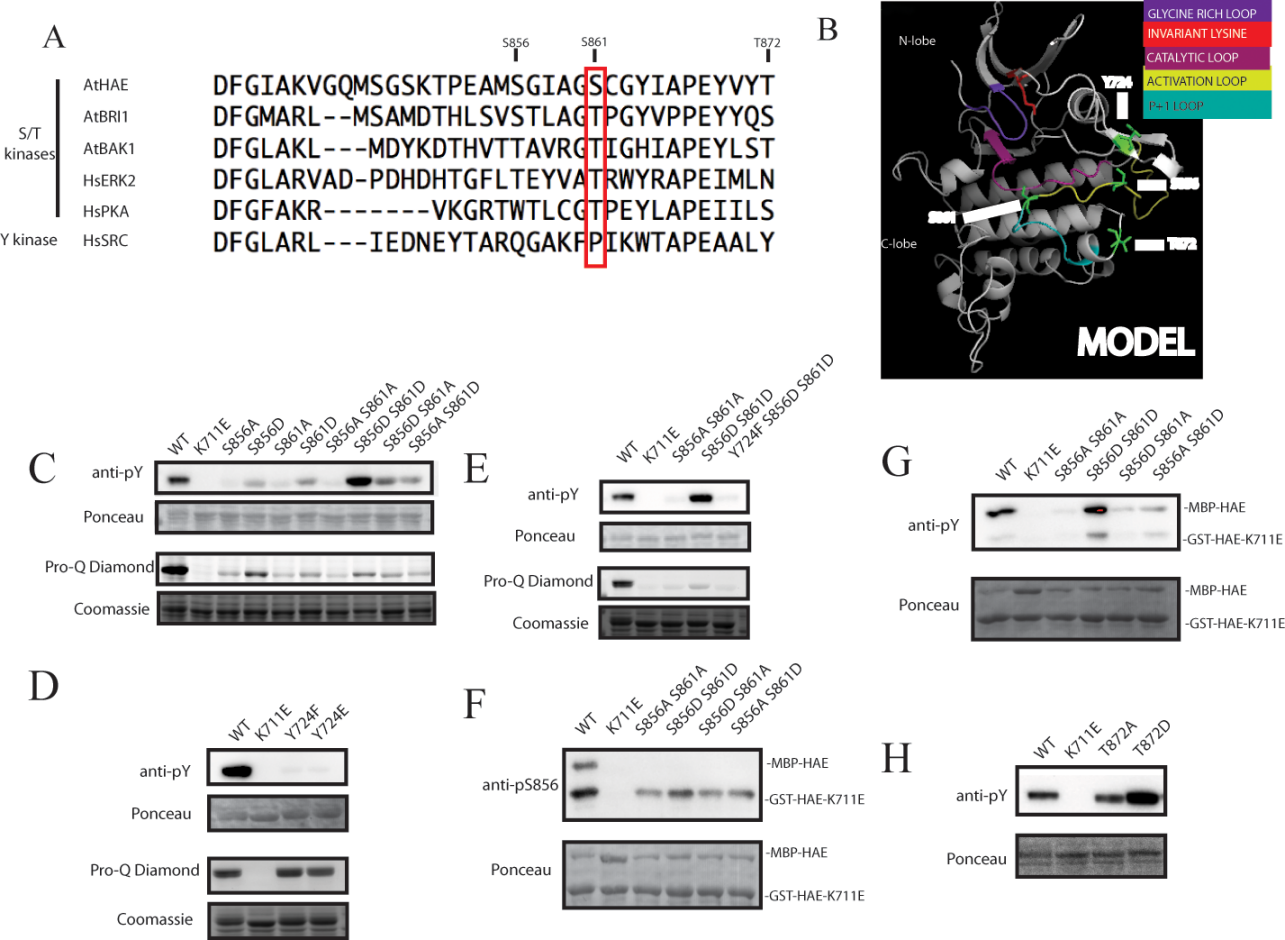
The double phospho-mimetic mutant S856D S861D exhibits enhanced tyrosine auto-phosphorylation *in vitro*. A) Alignment of the activation segment and C-terminal adjacent region of representative Arabidopsis RLKs and two human serine/threonine kinases and one human tyrosine kinase. B) Model of HAE protein kinase against a BRI1 crystal structure with Y724, S856, S861, and T872 displayed in green. C) Western blot with anti-pY antibody and Pro-Q Diamond stain analyzing recombinant MBP-HAE activation segment alanine and aspartate substitution mutants. D) Western blot with anti-pY antibody and Pro-Q Diamond stain analyzing recombinant MBP-HAE Y724F and MBP-HAE-Y724E. E) Western blot with anti-pY antibody and Pro-Q Diamond stain analyzing recombinant MBP-HAE S856D S861D substitution mutant in combination with Y724F mutation. F) Active MBP-HAE trans-phosphorylates inactive GST-HAE-K711E on S856 when co-expressed in *E*. *coli*. A substantial amount of MBP-HAE co-purifies with GST-HAE-K711E. G) Active MBP-HAE weakly trans-phosphorylates inactive GST-HAE-K711E on tyrosine when co-expressed in *E*. *coli*. H) The T872D phospho-mimetic mutant exhibits enhanced tyrosine auto-phosphorylation.

To contextualize these results, we examined the activation segment from the protein kinase complement of two well-studied eukaryotic organisms, human and Arabidopsis. Representative protein activation segment sequences, along with 5 residues C-terminal of the conserved APE motif, are aligned in Fig 7A. Included are 3 representative Arabidopsis RLKs, two human S/T kinases, and a human tyrosine kinase. Analysis of the primary sequence of human protein kinases, as well as literature focusing on non-plant eukaryotic protein kinases, shows that the position homologous to HAE-S861 and BAK1-T455 is a conserved serine or threonine in nearly all eukaryotic S/T kinases [29]. It lies at a hinge point that connects the two conserved loops that comprise the activation segment, the activation loop and P+1 loop [29]. Among RLKs, it has been shown that this residue is a conserved serine or threonine in over 99% of what are known as “RD kinases,” protein kinases containing a conserved arginine-aspartate motif in the catalytic loop [31]. Interestingly, only 30% of RLKs that have lost the RD motif (termed “non-RD kinases”) have retained a serine or threonine at this position, indicating a functional relationship between this residue and different modes of RLK catalysis [31]. We have recolored the model of the HAE protein kinase domain to indicate these structures, along with the location of S856 and S861 [Fig 7B]. The activation loop is the portion of the activation segment typically harboring activating phosphorylation sites, while the P+1 loop is involved in substrate interaction and stabilizing the C-terminus of the activation segment via interactions with the catalytic loop [29,30]. We hereafter refer to the conserved residue aligning to HAE-S861/BAK1-T455 as the “hinge serine/threonine.”

Contrary to the proposed emerging model with RLKs, in a number of protein kinase families the hinge serine/threonine has a well-established and essential function via the non-phosphorylated hydroxyl group participating in an interaction network with residues in the catalytic loop of the protein kinase [29]. This suggests two broad possibilities: the hinge residue has been co-opted to function as a phosphorylation site modulating activity in at least some RLKs, including HAE and BAK1; or alternatively, phosphorylation of S861 does not promote HAE activity, but rather the non-phosphorylated serine acts in accordance with traditional views of P+1 loop function to stabilize the activation segment-P+1 loop interaction. In this case, phosphorylation of the hinge residue on HAE could possibly function as a negative regulator of protein kinase activity by interfering with normal function of the hinge serine. This could explain the inability of the S861D and S856D S861D mutants to complement *hae hsl2*. These possibilities are discussed below.

In the BAK1 structure, while pT450 makes canonical interactions with multiple positively charged residues and overall exhibits characteristics of typical activation segment phosphorylation site, pT455 makes local interactions with adjacent residues in the P+1 loop that appear unique to this BAK1 structure, as well as an interaction with a conserved lysine in the catalytic loop. This interaction with the catalytic loop suggests a possible role of the phosphorylated hinge threonine in stabilizing the protein kinase in an active confirmation. As noted by Yan *et al*., despite widespread conservation of the hinge S/T in serine/threonine protein kinases, phosphorylation of this site may represent a novel activation mechanism because we are unaware of any other protein kinase crystal structure exhibiting phosphorylation at this site [44].

It is notable that the largest group of metazoan protein kinases that have substituted the conserved hinge S/T aligning to S861 are the tyrosine kinases, which possess a conserved proline at this position [29,56]. This is shown in the column aligning to HAE-S861 in Fig 7A, where the SRC tyrosine kinase possesses a proline at the hinge point between the activation and P+1 loops. Crystallographic analysis has shown that this proline is involved in contacting the phenolic group of the substrate tyrosine to position the residue for phosphorylation [57].

### The double aspartate substitution mutant S856D S861D displays elevated auto-phosphorylation of a single tyrosine residue

Given that phosphorylation of the residue homologous to S861 may be an RLK specific phenomenon, that tyrosine kinases have specifically substituted this residue to enable altered substrate specificity, and that RLKs have recently been shown to possess widespread dual specificity [32,34,58], we wondered whether phosphorylation at S861 may impact substrate preference of HAE toward serine/threonine and tyrosine residues. Additionally, during the course of our study, Bojar *et al*. published an analysis of the crystal structure of auto-phosphorylated BRI1 showing that the activation segment and adjacent regions of BRI1 exhibit distinct characteristics of both canonical S/T kinases, as well as tyrosine kinases, and suggested that differential phosphorylation of multiple residues on BRI1 may regulate substrate specificity, including the hinge residue BRI1-T1049 (homologous to HAE-S861), and BRI1-S1060 (homologous to HAE-T872) [42]. It should be noted that despite MS/MS evidence that the hinge residue BRI1-T1049 can be auto-phosphorylated *in vitro* on both the Arabidopsis and tomato orthologs of BRI1, the hinge residue (AtBRI1-T1049) was not shown to be a phosphorylation site on the analyzed BRI1 structures [42,45,59]. This suggests, but does not prove, that the hinge residue AtBRI1-T0149 is not a high occupancy *in vitro* auto-phosphorylation site of BRI1, potentially similar to HAE.

As a preliminary test of this hypothesis, we analyzed the MBP-HAE-S856/S861 activation segment single and double mutants by western blotting with an anti-phosphotyrosine antibody. Results of this experiment are displayed in Fig 7C. The wildtype protein kinase domain is readily detectable by this antibody, confirming work by Oh *et al*., as well as our MS/MS results, that at least *in vitro*, HAE exhibits dual specificity [32]. All other activation segment mutants display a reduction in signal except for the MBP-HAE-S856D S861D double mutant, which displays ~2.5 fold increase over wildtype, despite possessing only 20% level of auto-phosphorylation. This result suggests that the S856/S861 phospho-mimetic has altered preference toward tyrosine.

In the course of our *in vitro* phosphorylation site mapping, we positively identified a single phospho-tyrosine site, Y724. This tyrosine residue is predicted to reside in a long loop in the N-lobe of HAE [Fig 7B]. This loop does not appear to be conserved because it is not present in paralogous RLKs, including HSL1 and HSL2. To determine the extent to which this residue contributes to tyrosine phosphorylation of HAE, we mutated the site to encode non-phosphorylable phenylalanine, as well as to glutamate, a residue that partially mimics the negative charge, though not the structure, of phospho-tyrosine.

Results are displayed in Fig 7D. Mutation of this residue in wildtype resulted in nearly complete abolition of detection with the anti-pY antibody, suggesting Y724 is the major site of tyrosine auto-phosphorylation on HAE. Comparing the Y724F and Y724E mutants to wildtype indicates total auto-phosphorylation is essentially unaffected, suggesting that this tyrosine is not a major contributor to overall auto-phosphorylation, and that it is not essential for protein kinase activity [Fig 7D].

To test whether Y724 is similarly the major site of tyrosine auto-phosphorylation in the S856D S861D double mutant, we created the triple Y724F S856D S861D mutant of the MBP-HAE fusion protein and assayed auto-phosphorylation on tyrosine with the same anti-phopho-tyrosine antibody, as well as total auto-phosphorylation with Pro-Q Diamond. Results of this experiment show that tyrosine auto-phosphorylation is nearly completely eliminated in the triple Y724F S856D S861D mutant, indicating that Y724 is indeed the major site of tyrosine auto-phosphorylation [Fig 7E]. Analysis of total auto-phosphorylation with Pro-Q Diamond shows that the slight enhancement of auto-phosphorylation of the S856D S861D mutant over the S856A S861A mutant is abolished when Y724 is mutated [Fig 7E]. This result suggests that the increase in auto-phosphorylation of the S856D S861D mutant is nearly entirely attributable to phosphorylation of Y724.

We were next interested in whether we could determine if this site is auto-phosphorylated via an intramolecular or intermolecular mechanism. To test this, we co-expressed the inactive GST-HAE-K711E mutant from a modified pGEX vector with each of our activation segment mutants expressed as MBP-HAE fusions. As a control, we were able to show that HAE efficiently trans-phosphorylates GST-HAE-K711E on S856, using the phospho-specific pS856 antibody [Fig 7F]. Purification of the GST-HAE-K711E fusion from *E*. *coli* total protein yielded substantial amounts of MBP-HAE [Fig 7F]. This suggests the HAE protein kinase domain can stably interact with itself, as has been reported for other well-studied RLKs BRI1, BAK1, and SERK1 [42]. This results demonstrates that S856 can be phosphorylated by an intermolecular reaction, and in general that trans-phosphorylation is readily detectable using this coexpression assay. It is interesting that even activation segment mutants that display low levels of auto-phosphorylation and no complementation ability are able to transphosphorylate inactive HAE-K711E on the activation loop residue S856. Given that BRI1 activation segment phosphorylation sites do not especially conform to determined substrate preference motifs, it has recently been proposed BRI1 may utilize a mechanism of non-canonical phosphorylation called *activation segment exchange*, in which adjacent protein kinase molecules in a dimer swap activation segments to undergo phosphorylation on certain residues [36,60]. We similarly speculate HAE may utilize non-canonical mechanisms to achieve activation segment auto-phosphorylation.

Next we analyzed these samples for tyrosine trans-phosphorylation using the same anti-phospho-tyrosine antibody. Interestingly, there was relatively little tyrosine phosphorylation of the inactive K711E mutant [Fig 7G]. Much more signal was observed on the copurified MBP-HAE protein. Of the signal that was observed on the inactive mutant protein, the MBP-HAE-S856D S861D mutant consistently displayed the highest level of tyrosine trans-phosphorylation, suggesting it does in fact display higher levels of tyrosine phosphorylation trans-activity. However, further studies will be needed to determine if the lack of trans-phosphorylation is due to occurrence of Y724 auto-phosphorylation mainly as an intramolecular reaction, or whether the GST-HAE-K711E mutant N-lobe improperly conforms due to effects of the mutation of the conserved lysine K711.

One important additional observation is the double phospho-mimetic MBP-HAE-S856E S861E activation segment mutant displays drastically lower tyrosine auto-phosphorylation than double MBP-HAE-S856D S861D [S8 Fig]. This underscores the need for caution in interpreting the results of phospho-mimetic mutants *in vitro*. We further determined that a *HAE-YFP Y724F* mutant transgene expressed by the pGWB601 binary vector complements the abscission defect of *hae-3 hsl2*-3 at an efficiency similar to wildtype *HAE-YFP*, suggesting that this site is not essential for *in vivo* function [S9 Fig]. It may be that phosphorylation at this site does not regulate HAE activity. Alternatively, there are two serine residue immediately adjacent to Y724 (S725 and S726). It may be that phosphorylation at one or both of these sites coordinately contribute to regulation of activity, and that mutation of Y724 alone is insufficient to cause a phenotype.

Finally, in the analysis of crystalized BRI1 protein kinase domain, Bojar *et al*. noted that a conserved phosphorylated residue, BRI1-S1060, appeared to contribute to orienting the activation loop and speculated its phosphorylation might regulate protein kinase activity and interaction with substrates. This residue resides in a short helix adjacent to the activation segment and aligns to HAE-T872, which we have already shown can be phosphorylated on HAE *in vitro* and yields a protein kinase with a reduced level of auto-phosphorylation when mutated. To test whether the phospho-mimetic mutant of this residue exhibits altered tyrosine auto-phosphorylation, we analyzed the T872A and T872D mutants of the MBP-HAE fusion protein with the same anti-phospho-tyrosine antibody. Consistent with the hypothesis that phosphorylation of this residue impacts preference for tyrosine, the T872D mutant exhibits a ~2.5 fold increase in tyrosine auto-phosphorylation compared to both the wildtype protein kinase and T872A mutant [Fig 7H]. This result suggests phosphorylation of this conserved site C-terminal to the activation segment may impact HAE preference for tyrosine.

## Discussion

The ability of the S856A single mutant to partially complement the *hae hsl2* abscission deficient phenotype demonstrates phosphorylation of this residue is not absolutely required for HAE activity. However, three lines of evidence suggest phosphorylation of S856 nonetheless promotes HAE activity. First, *in vitro* evidence shows the MBP-HAE-S856D phosphomimetic mutant exhibits enhanced auto-phosphorylation over the non-phosphorylable MBP-HAE-S856A mutant. Second, the *HAEpr*:*HAE-S856A* mutant exhibits reduced activity *in vivo* over the *HAEpr*::*HAE* wildtype construct as measured by complementation efficiency of the abscission deficient phenotype of the *hae-3 hsl2-3* mutant. Finally, HAE and its close relatives all retain the conserved RD motif within the catalytic loop which correlates with a mode of protein kinase activation often, though not always, involving phosphorylation at or very near an activation loop site homologous to S856 [29]. HAE quite clearly exhibits partial function absent phosphorylation at S856, but phosphorylation at this site appears to enhance its activity. Therefore we infer that HAE has partially, though not completely, evolved away from a classical mode of RD protein kinase activation.

Evidence of positive regulation of HAE activity by phosphorylation of the activation loop residue S856 is considerable. In contrast, evidence of positive regulation of HAE activity by phosphorylation of the hinge residue S861 is largely circumstantial. It has been shown that mutation of the homologous residue in BRI1 and BAK1 heavily impairs protein kinase activity *in vitro*, and eliminates biological activity *in vivo* [22,31]. Mitra et al have also recently extended mutational analysis of the hinge residue to 3 additional Arabidopsis RLKs, encoded by the genes At2g01820, At2g01950 (BRL2), and At1g60800 (NIK3) [38]. This study found that alanine substitution of the hinge residue severely impairs protein kinase activity in two of the three protein kinases (encoded by At2g01820 and At2g01950). These results closely mirror those observed for HAE, where mutation of this residue *in vitro* or *in vivo* reduces HAE activity. An important difference between BRI1/BAK1 and HAE is that complete elimination of HAE complementation ability *in vivo* is only observed in the double S856A S861A mutant. While these results demonstrate convincingly that the hinge residue is important for function, alone they cannot differentiate between a general function of the hinge S/T in RLKs in a phosphorylated or non-phosphorylated state.

*In vitro*, it has been shown that phospho-mimetic mutants of BAK1 at the hinge threonine exhibit a slight increase in auto-phosphorylation as well as BRI1 trans-activation ability compared to the alanine mutant, suggesting that phosphorylation of this residue does increase BAK1 activity [22]. These results are reminiscent of those obtained with HAE phospho-mimetic mutants of the hinge serine, where the aspartate substitution mutants displays higher auto-phosphorylation than the corresponding alanine mutants. In the case of HAE, however, it appears the most or all of the enhanced auto-phosphorylation is due to hyper-phosphorylation of a single tyrosine residue [discussed below]. To our knowledge, the relative contribution of tyrosine phosphorylation activity of BAK1 in the hinge residue phospho-mimetic mutants has not been examined.

Despite an apparent, slight increase in activity of the hinge residue phospho-mimetic mutants of BAK1 and HAE *in vitro* compared to the alanine mutants, these corresponding hinge residue phospho-mimetic mutants of HAE, BAK1, and BRI1 are completely non-functional in complementation assays *in planta* [22,31]. These results could imply that phosphorylation at this site does not generally activate the protein kinases, or the non-complementation may be due to interference with normal protein function by the phospho-mimetic mutation in comparison to the genuine phosphorylated hinge residue. A third possibility is that variable phosphorylation of this residue directs the protein kinases to different substrates and that locking the protein kinases into the phosphorylated state with a phospho-mimetic mutation may preclude some essential substrate phosphorylation.

To our knowledge, no other protein kinase has been shown to be activated by phosphorylation at the hinge reside, despite conservation of a phosphorylable serine or threonine at this position in almost all S/T kinases. One important exception to this pattern of conservation is observed in non-RD plant RLKs [31]. Outside of plant protein kinases, this residue is generally thought to function in the non-phosphorylated state in substrate binding and by its interactions with conserved residues in the catalytic loop. The repeated identification of this site as an *in vitro* phospho-site of RLKs, albeit at a low level in the case of HAE, as well as the *in vivo* identification of this phosphorylation site on BAK1, does suggest functionality. However, we cannot conclude from phospho-site identification alone what the nature of this functionality may be, whether by positive or negative regulation. For example, it has recently been shown that *in vitro*, phosphorylation of the homologous hinge threonine in the Arabidopsis MKK4 MAP kinase kinase correlates with reduced activity toward MAP kinase substrates, suggesting that phosphorylation of this residue may negatively regulate MKK4 [61]. This result demonstrates that we cannot extrapolate a mode of regulation (positive or negative) on the basis of phospho-site identification alone. Nonetheless, the fact that RLKs of the RD class retain a phosphorylable residue at this position, while those of the non-RD class do not, is very suggestive of the possibility that this residue functions in the regulation of RLKs.

An important recent study analyzed the structure of a BAK1 crystal containing high levels of auto-phosphorylation at the hinge threonine, T455 [44]. As noted by the authors, the hinge residue phosphate is engaged in a hydrogen bond with a conserved lysine within the catalytic loop, which is a characteristic behavior of the non-phosphorylated hinge residue hydroxyl in active S/T kinases [29,44]. This result suggests a mechanism by which the phosphorylated hinge threonine in BAK1, and possibly other RLKs, is at least partially functionally equivalent to non-phosphorylated S/T hinge residue in other S/T kinases. This finding may help bridge our understanding of the way in which specific activation mechanisms of RLKs have diverged from other protein kinases. An alternative model is that phosphorylation of the hinge residue is involved in negative auto-regulation. Inhibitory auto-phosphorylation has recently shown in studies of BRI1 [62].

Regardless of whether S861 is a phosphorylation site, the ability of the S861A single mutant to abscise, but not the S856A S861A double mutant, suggests that HAE can still be activated in an S856-dependant manner. Based on the strength of the evidence that phosphorylation of S856 functions as a positive regulator of HAE activity, and on the extent to which the S861A mutant is impaired *in vitro*, we hypothesize that the S861A mutant protein can be trans-phosphorylated on S856 by the activity of a trans-activating co-receptor protein kinase, similar to a model such as that of BRI1/BAK1 activation. BAK1 is a member of the SERK family of co-receptor protein kinases whose best studied function is as trans-activating co-receptors in hetero-oligomeric complexes in which they activate the protein kinase domain of their ligand binding RLK partners [63]. A member of the SERK family, *SERK1*, has genetically been shown to regulate abscission, but it is thought to be a negative regulator of the process, based on the ability of *serk1* mutants to suppress the abscission defect of the *nevershed* mutant [64,65]. It is possible SERK1 has a complex role in abscission and also positively regulates HAE activity, or there could be another SERK protein or a novel trans-activating protein kinase that activates HAE.

One interesting and unanticipated result of this study is the fact that very low levels of *HAE-YFP* transgene expression are required to complement the abscission defect of *hae-3 hsl2-3*. This is true of both *HAE-YFP* wildtype and the partially impaired mutants *HAE-YFP S856A*, *HAE-YFP S856D*, and *HAE-YFP S861A*. These results suggests that HAE may exist in excess of other essential signaling components (such as ligand, co-receptors, and/or substrates). As additional knowledge is gained about the composition of the HAE protein complex that activates abscission signaling, as well as downstream signaling components, it will be interesting to study why such a wide wide-range of HAE dosage is apparently capable of activating abscission.

The mechanism of RLK dual specificity has not been established. Work from Oh *et al*. showed that *in situ*, BRI1 tyrosine auto-phosphorylation accelerates as expression and incubation time increase, concluding there may be phosphorylation sites that induce a substrate specificity switch or relaxation [37]. Analysis of the BRI1 crystal structure by Bojar *et al*. suggests two regions that may be candidates for phospho-regulation of substrate specificity as the P+1 loop, and the region C-terminal to the activation segment near an adjacent alpha helix [42].

It is interesting that some, though not all, phospho-mimetic mutants of HAE at these predicted sites enhance auto-phosphorylation of tyrosine. It is not presently known if tyrosine phosphorylation has any role in regulating HAE activity or whether HAE tyrosine phosphorylates any substrate. The ability of the *HAE-YFP Y724F* mutant to complement *hae-3 hsl2-3* clearly demonstrates it is not an essential phosphorylation site for activity. Additional HAE *in vivo* phosphorylation site mapping, mutational analysis, and identification of *in vivo* substrates will be highly informative in understanding what role, if any, tyrosine phosphorylation plays in regulating HAE activity.

Still, anecdotal evidence points to evolution of the P+1 loop hinge residue as playing an integral role in the evolution of tyrosine phosphorylation activity in multiple independent protein kinase lineages. Substitution of this residue by proline is a major structural contributor to metazoan tyrosine kinase specificity. Furthermore, the non-plant eukaryotic WEE family of atypical tyrosine/dual-specificity protein kinases exhibits substitution of the hinge residue with aspartate [66]. If it is true that differential phosphorylation alters substrate specificity or preference, it implies RLKs have multiple signaling modes that may allow increased complexity of information readouts from the plasma membrane. Such phosphorylation regulated dual specificity could occur by a number of mechanisms. It may cause a substrate specificity switch, from serine/threonine to tyrosine residues, via alterations of the substrate binding properties of the P+1 loop and adjacent helix. It may also cause a substrate preference relaxation, where the protein kinase simply loses specificity, depending on the pattern of its phosphorylation. Additional work on other model RLKs will help determine whether this proposed mechanism reflects a general mechanism to achieve dual specificity.

## Model

**Fig 8:**
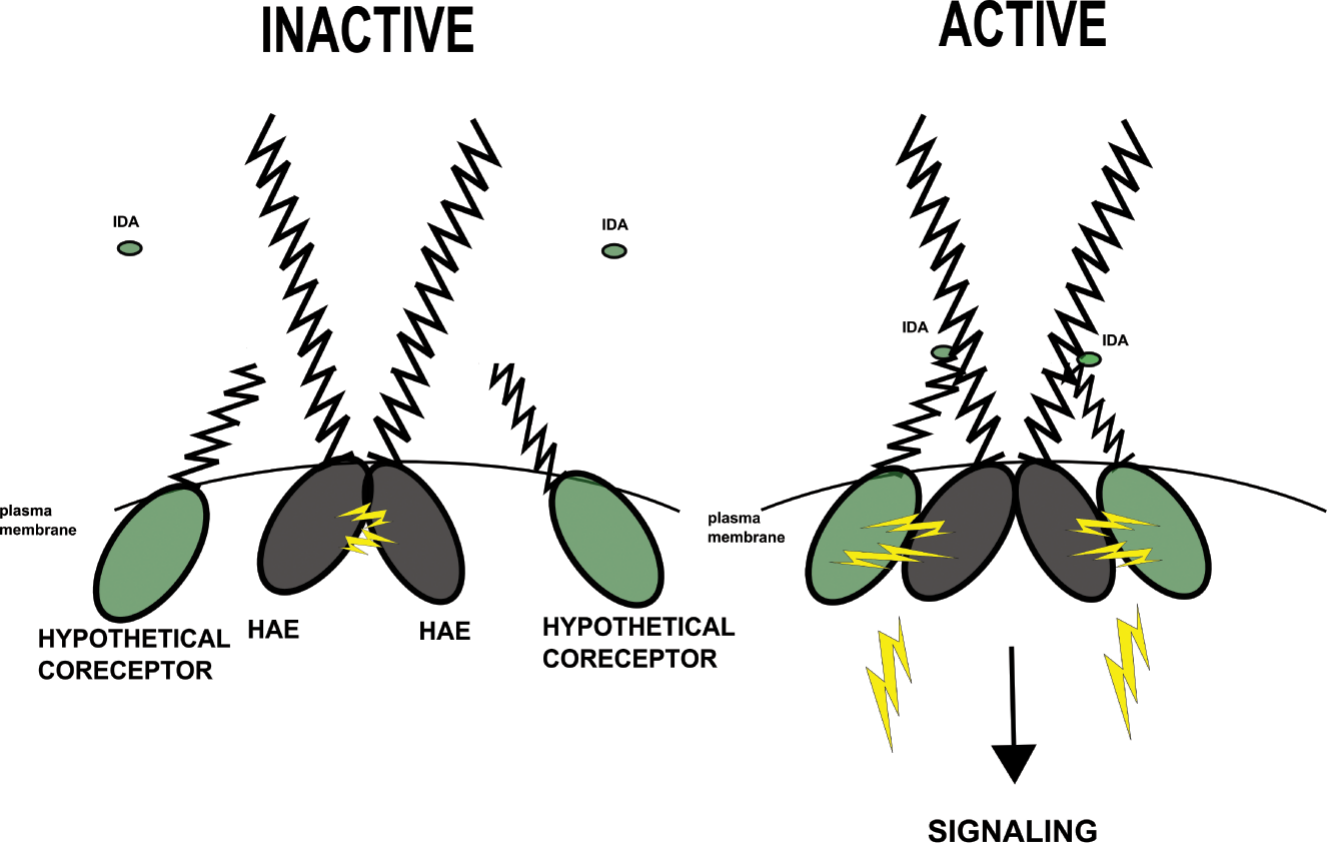
Hypothetical model of HAE activation.

A major property of protein kinases is that they can be conditionally activated. One important finding of this work is that phosphorylation at the primary site S856 is not the activating switch responsible for converting HAE from the inactive to active state, as evidenced by the ability of S856A mutant to partially complement *hae hsl2*. This is perhaps not surprising given that HAE and other RLKs are receptor proteins. Instead, we hypothesize perception of ligand has evolved to be the activating switch of HAE and related LRR-RLKs. This hypothesis largely stems from well-supported activation mechanisms of the LRR-RLKs BRI1, FLS2, and EFR, which have been shown to undergo ligand induced association with co-receptors of the SERK family of LRR-RLKs [15,16,23,24,31]. We hypothesize that HAE functions in a similar manner. In this model, inactive HAE auto-associates, possibly exhibiting constitutive phosphorylation on S856 We hypothesize this association is mediated by the HAE intracellular protein kinase domain due to the results of this study indicating it has the ability to auto-associate *in vitro*. Binding of the IDA derived ligand induces high affinity association with the extracellular domain of a trans-activating co-receptor protein kinase, which brings the heteromeric protein kinase domains into proximity, possibly leading to phosphorylation of S861, as well as S856 residues that had not been auto-phosphorylated, full protein kinase activation, and access to signaling substrates [Fig 8].

## Conclusion

This study provides a foundation upon which to understand HAE activity, and defines several avenues of future research, both specific to HAE and generally to RLKs. *In vivo* phosphorylation site mapping and identification of signaling substrates and putative activating co-receptors are clear objectives, and will lead to additional gains in our understanding of the activation of HAE and propagation of signals during abscission. Work from this study demonstrates that S856 is likely an activating phosphorylation site. The lingering ambiguity of the function of S861 and homologous RLK residues is still in need of resolution, and additional work is needed to certify what specific effect phosphorylation of this residue has on HAE specifically, and RLKs generally. The finding that various phospho-mimetic HAE mutants exhibit increased preference for tyrosine *in vitro* suggests phosphorylation may play a role in shifting substrate specificity of RLKs. Further work on HAE and other RLKs is needed to test and refine this hypothesis. Finally, this work demonstrates that abscission is a sensitive phenotypic outcome to test hypotheses regarding determinants of RLK function.

## Materials and Methods

### Plant growth conditions

Plants were grown in a 16 hour light cycle at ~700 lux, 20°C during light, 18°C during dark, between 35 and 75% humidity. Plants were fertilized with Miracle-Gro diluted in water to a concentration of 120 ppm N ~every two weeks.

### Cloning and mutagenesis

The *HAEpr*::*HAE-YFP* construct was created by ligating a 4.7 kb PCR fragment including the upstream 1.6 kb of HAE and the entire HAE gene up to and excluding the stop codon into the BamHI/NotI sites of the gateway entry vector pE6c [67]. Mutagenesis of this plasmid to create various mutants was performed by a combination of the Quickchange mutagenesis protocol and PCR based self-ligation mutagenesis as indicated in the primer table [68] [S2 Table]. All constructs were Sanger sequenced across the HAE coding region and YFP tag using primers listed in S2 Table. Entry vectors were inserted into the binary vectors pBIB-GWR-BASTA or pGWB601 using Gateway recombination as indicated in the text [69][70]. 35S::HAE-GFP was created by cloning the HAE coding region into pBIB-35S-GFP-BASTA via a KpnI site. Constructs were transformed into Arabidopsis via floral dip [71].

For indicated experiments, we utilized the pGWB601 binary vector, instead of pBIB-Basta-GWR, which we found has a more convenient bacterial selectable marker (SpecR, versus KanR in pBIB-Basta-GWR, which allows easier selection of recombinants with pENTR-KanR derivatives) and higher cloning efficiency. However, we subsequently found HAE-YFP constructs expressed via pGWB601 have a slightly lower overall complementation ability than HAE-YFP constructs expressed via pBIB-Basta-GWR (~77% for pGWB601, compared to ~97% for pBIB, Figure A in S10 Fig). We hypothesize the proximity of the strong promoter driving expression of the *BAR* gene in pBIB-Basta-GWR near the 5’ end of the HAE promoter may create an open chromatin confirmation that leads to fewer non-expressing T1 lines, in comparison to the end to end configuration of the pGWB601 binary vector [Figure B in S10 Fig]. Effects of binary vector structure have previously been shown to affect transgene expression [72].

pMAL-HAE-KD and pGEX-HAE-KD were previously described [25,26]. Mutagenesis of these plasmids was performed by a combination of the Quickchange mutagenesis protocol and PCR based self-ligation mutagenesis as indicated in S2 Table. pGEX-HAE-KD K711E SpecR was created by blunt end ligation of a polynucleotide kinase treated PCR fragment of the spectinomycin gene from pCR8 (Life Technologies, Carlsbad, CA) into a PCR amplified, DpnI treated pGEX-HAE-KD K711E fragment with the ampicillin resistance gene deleted by PCR. All constructs were Sanger sequence verified.

### Purification of *in situ* auto-phosphorylated HAE intracellular domain

For purification of GST-HAE, 3 ml of an overnight culture of pGEX-HAE containing *E*. *coli* strain BL21 (DE3) was transferred to 50ml liquid LB medium containing 100μg/ml ampicillin and grown at 37 C to a density of about 0.8 OD_600_. IPTG was added to 0.1mM and cells were grown overnight at 22 C. The cells were collected by centrifugation, washed by lysis buffer (50 mM HEPES, pH7.4, 150 mM NaCl, 10 mM EDTA with freshly added 10 mM PMSF) and lysed via sonication for 3 min. The insoluble material was removed by centrifugation at 12,000 x g 30 min. The clear extract was added to 5 ml of a 10% w/v solution of the glutathione-agarose and incubated for 30 min at 4°C. The beads were collected and washed three times with 10 ml of lysis buffer. The recombinant protein was eluted by adding a solution containing 15 mM glutathione in 50 mM HEPES, pH 7.4. MBP-HAE was purified in a similar manner, except cultures were grown at 22.5°C for 5.5 hours for replicate 1 or overnight for repliacate 2, and .4 ml bed volume of amylose resin in PBS was used for batch affinity purification with 2 hour incubation, and 10 mM maltose in PBS was used in elution.

### Intact mass analysis

Intact mass analysis of GST-HAE and MBP-HAE-T851A was performed by diluting the sample in 1% formic acid to pmol/μL immediately before analyses. Following centrifugation to remove any precipitated material, a portion of this dilution (10 μL) was placed in a polypropylene autosampler vial and placed in the autosampler cooled to 4°C. A short LC-MS run was used to analyze the samples as follows: a 1 μL (or 0.5, or 0.1 μL) aliquot was injected onto an Agilent C8 column (Zorbax C8, 43mm long, 75um inner diameter, Agilent cat# G4240-63001 SPQ105). An LCMS run with the following parameters was used: acquired mass range 300-2500m/z; 0.63 spectra/second; fragmentor at 250V or 200V. Gradient as follows: initial conditions (for trap load) was 3%B (A: 0.1% formic acid in water; B: 99.9% acetonitrile, 0.1% FA); rapid ramp to 20%B over 1 min; hold at 20%B for 3 min; gradient 20–60%B over 6 min; ramp to 90%B over 1min; hold at 90%B for 4 min; ramp back to initial conditions over 30 sec and hold at 3%B for 3 min prior to loading next sample. Total run time was 18.5 min. The data were then examined using the qualitative analysis software provided with the instrument. Using data from the major peak in the chromatogram (TIC-total ion current), protein masses were deconvoluted from their multiply-charged species present. Default values for deconvolution were used.

### Phospho-peptide enrichment and phospho-site identification

For GST-HAE phosphorylation site mapping, purified GST-HAE was acetone precipitated and the pellet was resuspended at 1 μg/μL in 6 M urea, 100 mM HEPES, pH 8.0, reduced (DTT) and alkylated (IAA), and digested separately with trypsin or Glu-C overnight at 37°C or 25°C, respectively. Digests were acidified (formic acid to 1% v/v) and then analyzed on the Agilent 6520 Accurate Mass QTOF using their phospho-chip column (Agilent Technologies Inc., catalogue # G4240-62021). This phospho-chip column has a 40nL tri-partite trap column consisting of C18-TiO2-C18 and a 43mm C18 analytical column that facilitates analysis of non-phosphorylated peptides and TiO2-enriched phospho-peptides. Two LCMS runs were to identify non-phosphorylated peptides, followed by a second (shorter) gradient to identify TiO2-enriched phospho-peptides.

For the first MBP-HAE-T851A replicate, 50 μg of purified protein was precipitated with acetone to remove PBS. Acetone pellet was resuspended in 10 μL in 6M urea, 100mM HEPES, pH 7.8. An in-solution trypsin digest of the sample was conducted as described above. Following digest, the sample was acidified and subjected to large-format C18 tip (Pierce-ThermoFisher) purification with peptides being eluted with 80% acetonitrile, 2% formic acid, in water. Half the sample was lyophilized and analyzed on the Agilent QTOF using the phospho-chip using the same conditions as the GST-HAE above. The other half was loaded onto a TiO2 tip (Glygen Corp, NT2TIO.96) and eluted with 25 μL pH 11 ammonium phosphate. Both TiO2-enriched and the “TiO2 tip-exposed” fractions were lyophilized and acquired separately on the LTQ Orbitrap XL using a neutral-loss multi-stage activation (NL-MSA) LCMS method. Briefly, this method examined MS/MS (MS2) spectra for “neutral losses” of the phosphate moiety from Ser/Thr-phosphorylated peptides. Once a neutral loss is found, a second round of MS/MS (MS3) is triggered on the neutral loss peak. These two spectra (MS2 and MS3) are automatically combined into a single merged spectrum. Peptides were loaded directly onto a 25cm x 150um pulled-needle analytical column packed with HxSIL C18 reversed phase resin (The Hamilton Co.). A step gradient of acetonitrile was used to elute peptides at 400 nL/min over a total run time of 110 minutes.

For the second MBP-HAE-T851A replicate, 225 μg of purified protein was precipitated with acetone to remove PBS. Acetone pellet was resuspended in 20 μL in 6M urea, 100 mM HEPES, pH 7.8 and split in two. Half was subjected to an in-solution trypsin digest and the other was subjected to in-solution Glu-C digestion. Following digest the samples were acidified, subjected to large-format C18 tip purification, and NL-MSA LCMS on the LTQ Orbitrap as described above.

All data were searched against two databases: the NCBInr limited to Viridiplantae database (2,355,794 entries, last update 12/6/2013) or the protein of interest, MBP-HAE-T851A, concatentated with the cRAP DB (113 entries, last update 4/15/2015). Database searches were conducted using the Sorcerer2 Integrated Data Appliance (SageN Research). Criteria for searches included: trypsin, 2 missed cleavages allowed, carbamidomethyl-Cys (fixed), oxidized Met and phosphoSTY (differential) modifications, 25 ppm mass error allowed. Data were then examined using Scaffold V3.4.8 software. Further data filters applied included 99% confidence on protein ID, 95% confidence on peptide match, <10 ppm mass error filter. Additionally, manual validation of phospho-sites was conducted by examining individual MSMS spectra.

### *In situ* auto-phosphorylation assay

We performed the mutagenesis and *in situ* auto-phosphorylation assays on a plasmid encoding MALTOSE BINDING PROTEIN/MBP-HAE due to the significantly higher accumulation of protein we observed compared to GST-HAE in total cell lysate [S11 Fig]. We reasoned that as both fusion proteins are known to auto-phosphorylate *in vitro* [25], phospho-sites critical for protein kinase function will not vary between the two.

*In situ* auto-phosphorylation reactions as previously described [26]. In brief, we inoculated overnight bacterial cultures of *E*. *coli* strain BL21 containing each mutant into fresh media and grew to an OD_600_ of between .6–.8, then induced expression by addition of IPTG to a final concentration of .1 mM for 4 hours at 25°C at 300 RPM shaking. 100 μL of each culture was then spun down, resuspended in 100 μL of 1x SDS samples buffer, and boiled for 5 minutes. The samples were then separated by SDS-PAGE on a mini-gel followed by sequential staining with 15 ml of 1/3^rd^ strength Pro-Q Diamond according to the method of Agrawal and Thelen [73], and Coomassie Blue Silver stained [74]. Images of Pro-Q Diamond fluorescence were taken with a BioRad Chemidoc using default settings, and Coomassie staining with a BioRad Geldoc. The Pro-Q Diamond/total protein signal ratio was calculated by subtracting the background signal for a sample of empty vector lane from both quantities, then normalizing the ratio of each sample wildtype, which was defined as having ratio 1.

Because of the limitation of 15 lanes on a standard minigel and the difficulty in controlling for minor gel to gel variability in staining, we analyzed the initial 9 alanine mutants by comparing the ratio of their auto-phosphorylation levels to a wildtype sample run on the same gel. 4 gels were analyzed this way with 4 sets of biological replicates. This yielded a set of 4 mutant/wildtype ratios for each mutant. These ratios were analyzed by a one-sample two-tailed T-test, comparing the set of ratios of mutant/wildtype to 1 (the expected ratio if the null hypothesis of no difference was true). Single alanine to aspartate comparisons were performed on a single gel each, allowing comparison of 3 wildtype samples to 3 alanine mutant and 3 aspartate mutant samples. The averages of the auto-phosphorylation levels for each construct were analyzed with a two-sample two-tailed T-test, assuming unequal variance. Analysis of the activation segment mutants was performed by running 4 biological replicates sets of alanine and aspartate mutants along with a wildtype control on 4 different gels, as before. Single mutants of the same residue were compared with a two-sample, two-tailed T-test assuming unequal variance. Comparisons between double activation segment residues was performed by ANOVA with Tukey-Kramer correction for multiple comparisons.

### Microscopy and image processing

Fluorescence of YFP and GFP protein in Arabidopsis flowers in different stages were examined either under a Leica MZFLIII stereoscope coupled to a 12-bit color CCD camera with images processed with the ImageQ software (Leica), or a Zeiss Discovery.V12 fluorescent stereoscope with Canon EO5 6D camera with images processed by Adobe Photoshop.

### Transgenic characterization and phenotyping

Petals breakstrength measurements were taken using a petal breakstrength meter as described [75]. The values for the petal breakstrength from each indicated floral position were analyzed from 15 plants from two independent transgenic lines for both wild type and mutant constructs.

A single representative YFP expressing T1 individual for each transgenic construct was selected for sequence validation by PCR across the expected mutation site/sites and Sanger sequencing utilizing a primer pair flanking the *HAE/YFP* junction. Genotyping for the correct parental mutant genotype was carried out by analysis with a *hsl2-3* dCAPS marker for *hae-3 hsl2-3*, or a *hsl2-4* dCAPS marker for the *hae-5 hsl2-4* mutant [41].

For mutants displaying complete lack of abscission (S856A S861A, S861D, S856D S861D) the entire HAE coding sequence of the HAE transgene was sequenced by PCR amplification with *HAEpr/YFP* specific primers, and subsequent Sanger sequencing analysis with primers listed in S2 Table, in order to rule out the possibility of the introduction of spurious mutations during subcloning.

Qualitative phenotyping was carried out by lightly brushing the inflorescence of the indicated number of T1 individuals to remove detached floral organs lightly stuck to the developing siliques. In Arabidopsis, the first flower post-anthesis is defined as position 1, and each older flower is numbered sequentially. Individuals were scored as “full abscission” if all flower positions beyond position 10 showed complete abscission, “partial abscission” if any number of floral organs beyond position 10 retained floral organs, and “abscission deficient” if no floral organs obviously abscised.

### Phylogenetic analysis and comparative phosphorylation site identification

We iteratively searched the Google Scholar Database with the following terms:

“autophosphorlyation receptor-like kinases plants,” “autophosphorlyation receptor-like kinases arabidopsis,” “autophosphorlyation site mapping receptor-like kinases plants,” autophosphorlyation site mapping receptor-like kinases arabidopsis.”

Publications were included in the analysis after it was determined they contained evidence of specific phosphorylation sites. We determined we had reached near saturation by repeated failure to find unindexed reports with additional search terms. Sequences for proteins from each study were downloaded from UniProt, aligned by ClustalW, and the activation segment region from the conserved DFG to APE motifs were exported to a subalignment. Publications were then analyzed for reports of phosphorylation on residues lying within this activation segment sub-domain.

### Co-expression and western blotting

#### Anti-pS856 *in vitro* validation

The polyclonal anti-phosphopeptide antibody was raised in rabbit against the peptide CSGSKTPEAMpSGIAGS (where pS corresponds to pS856). The anti-pS856 antibody was generated and affinity purified by Genscript (Piscataway, NJ).

To determine phospho-specificity of the anti-pS856 antibody, 5 μl of total *in situ* phosphorylated wildtype and protein kinase-dead MBP-HAE were separated by SDS-PAGE alongside an empty vector control, blotted to a nitrocellulose membrane, stained with Ponceau-S, imaged, blocked with 4% BSA in PBS with 0.1% Tween-20 (PBS-T), probed with 1:500 anti-pS856 overnight at 4°C, rinsed 4x with PBS-T for 20 minutes total, incubated 1 hour at room temperature with 1:2500 dilution of anti-rabbit-HRP (Cell Signaling Technologies, Danvers, MA), rinsed 4x with PBS-T for 20 minutes total, incubated with chemiluminescent substrate (Super Signal West Pico, Life Technologies, Carlsbad, CA), and imaged with a BioRad Chemidoc.

#### Anti-pS856 *in vivo* validation

Detection of pS856 *in vivo* was accomplished by generating total microsomal membrane preparations from whole stage 15 flowers of Col, *hae-3 hsl2-3*, and a complemented *hae-3 hsl2-3 35S*::*HAE-GFP* line, homogenized with 10 mM HEPES-KOH buffer (pH 7.5), containing 250 mM sucrose, 5 mM EDTA, 5 mM EGTA, 1 mM PMSF, protease inhibitor cocktail (10 μg/ml leupeptin, 5 μg/ml chymostatin, and 10μg/ml aprotinin), and centrifuged at 8000g for 15 min. The resultant supernatant was centrifuged at 100,000g for 30 min to separate the total membrane fraction (pellet). All manipulations were carried out at 4°C. pS856 was detected as before except with a film imager for enhanced sensitivity.

#### Tyrosine phoshporylation detection and co-expression

Detection of tyrosine phosphorylation on recombinant MBP-HAE was accomplished identically as above, using an anti-pY primary antibody at 1:5,000 dilution (clone 4G10, EMD Millipore, Billerica, MA), and anti-Mouse-HRP (Cell Signaling Technologies) secondary antibody.

#### Coexpression

Coexpression and affinity isolation of GST-HAE/MBP-HAE was accomplished by batch affinity purification of GST-HAE-K711E as above from 10 ml cultures of the GST-HAE-K711E/MBP-HAE co-transformed and co-expressing cultures of *E*. *coli* strain BL21. These strains were creating by electroporating mid-log phase pGEX-GST-HAE-K711E-SpecR containing *E*. *coli* strain BL21 with the respective pMAL-MBP-HAE-AmpR plasmids before selection on LB-Agar plates containing 50 μg/ml of both, Spectinomycin and Ampicillin. Two single colonies were picked for each transformation and analyzed. Expression was carried out as before. Western blots analyzing tyrosine phosphorylation and S856 phosphorylation of the co-expressed proteins were carried out as above.

## Supporting Information

S1 Fig Gene model of HAE and HSL2 mutant alleles used in this study. “TM” refers to the transmembrane region.
(TIF)

S2 Fig Intact mass of *in situ* auto-phosphorylated GST-HAE. A) Indicated peaks refer to calculated mass of respective number of phosphate groups on GST-HAE. Secondary peaks correspond to isoforms with undetermined modifications, presumably from E. coli enzymes. B) Intact mass of MBP-HAE-T851A replicate 1. C) Comparison of total auto-phosphorylation between MBP-HAE-T851A replicate 1 and 2.
(TIF)

S3 Fig 35S::HAE-GFP complements the abscission deficient phenotype of *hae-3 hsl2-3*. (TIF)

S4 Fig Partial complementation of *hae-3 hsl2-3* by *HAEpr*::*HAE-YFP S856A* transgene. A) Full complementation and non-rescued phenotypes and YFP accumulation of individual T1 plants expressing *HAEpr*::*HAE-YFP wildtype*. Arrows point to the abscission zone in fluorescent photographs of weakly expressing and non-expressing lines. B) Representative fully, partially, and non-complemented *HAE-YFP S856A* T1 lines. Red arrows indicate non-abscised floral organs. C) Abscission phenotype and YFP accumulation for representative fully and partially rescued T1 lines of *HAE-YFP S856A*. Arrows point to the abscission zone in fluorescent photographs of weakly expressing lines. D) Abscission phenotype and YFP accumulation for representative non-rescued T1 line of *HAE-YFP S856A*. Arrow points to the abscission zone in fluorescent photograph of a non-expressing line.
(TIF)

S5 Fig *HAEpr*::*HAE S856A* can partially complement *hae hsl2* irrespective of presence of YFP tag or allelic background of *hae hsl2* mutant. A) Representative plant of *HAE-S856A (YFP truncation)* in *hae-3 hsl2-3*. B) Representative T1 plant of *HAE-YFP* in *hae-5 hsl2-4*.
(TIF)

S6 Fig *HAE-YFP S856A vs S856D* complementation of *hae-3 hsl2-3*. A) Partial and full complementation phenotypes of individual T1 plants expressing *HAEpr*::*HAE-YFP S856A* and *S856D* transgenes. B) Abscission phenotype and YFP accumulation for representative fully and partially rescued T1 lines of *HAE-YFP S856D*. Arrows point to the abscission zone in fluorescent photographs of weakly expressing lines. C) Abscission phenotype and YFP accumulation for representative non-rescued T1 line of *HAE-YFP S856D*. Arrow points to the abscission zone in fluorescent photographs of a non-expressing line.
(TIF)

S7 Fig YFP accumulation in *HAE-YFP* activation segment single and double mutant T1 lines. A) Abscission phenotype and YFP accumulation for representative fully and partially rescued T1 lines of *HAE-YFP S861A*. Arrows point to the abscission zone in fluorescent photographs of weakly expressing lines. B) Abscission phenotype and YFP accumulation for representative non-rescued T1 line of *HAE-YFP S861A*. Arrow points to the abscission zone in fluorescent photographs of a non-expressing line. C) YFP accumulation in representative T1 lines for the *HAE-YFP S856A S861A*, *HAE-YFP S861D*, and *HAE-YFP S856D S861D* mutants.
(TIF)

S8 Fig MBP-HAE-S856E S861E exhibits much lower tyrosine auto-phosphorylation than MBP-HAE-S856D S861D. (TIF)

S9 Fig The *HAE-YFP Y724F* mutant efficiently complements *hae-3 hsl2-3*. (TIF)

S10 Fig Complementation efficiency and structure of two Gateway compatible binary vectors used in this study. A) A population of pBIB-Basta-GWR HAEpr::HAE-YFP and pGWB601 HAEpr::HAE-YFP T1 transformants was grown side by side to compare complementation efficiency. 44/44 pBIB T1 plants fully abscised, whereas 20/26 pGWB601 T1 plants fully abscised. B) Structure of binary vectors. Arrow labelled “*HAEpr*::*HAE-YFP*” indicates direction of recombination from 5’->3’ end of HAE transgene.
(TIF)

S11 Fig Protein accumulation of GST-HAE and MBP-HAE in total *E*. *coli* cell lysates after 4 hour induction period. Coomassie stained gels of total cell lysates from GST-HAE and MBP-HAE expressing *E*. *coli*. Intervening lanes were deleted from the GST-HAE gel.
(TIF)

S1 Table MS/MS data. (XLSX)

S2 Table Primer sequences. (XLSX)
