# Supplementary figures and images for: Analysis of Phosphorylation of the Receptor-Like Protein Kinase HAESA during Arabidopsis Floral Abscission
Source: PLoS One. 2016 Jan 19;11(1):e0147203. doi: 10.1371/journal.pone.0147203 (PMC4718614; doi:10.1371/journal.pone.0147203)

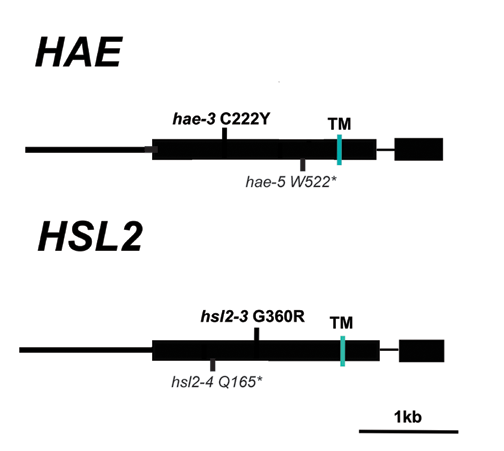

Supplement: S1 Fig — “TM” refers to the transmembrane region. (TIF) [file pone.0147203.s001.tif]

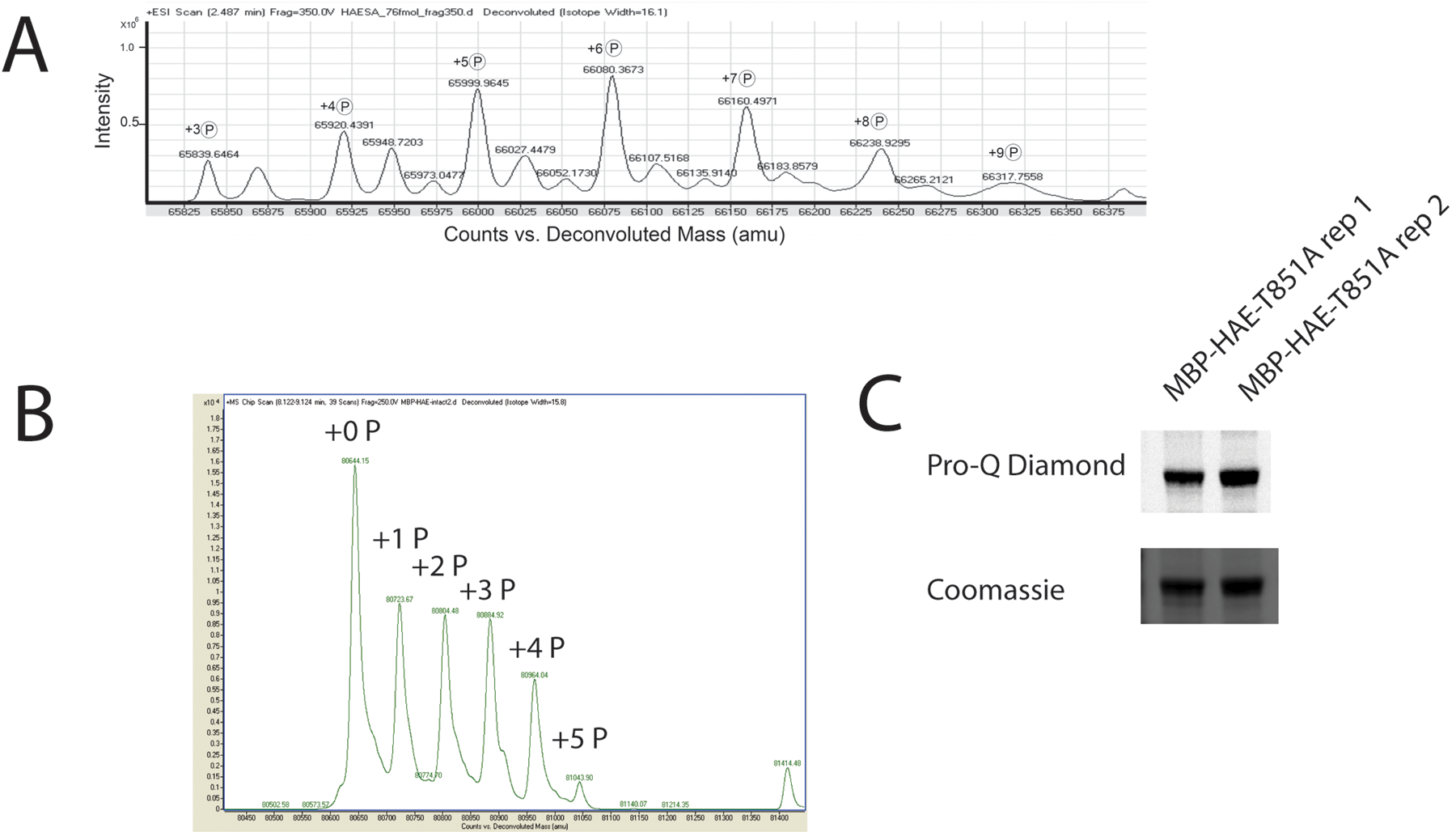

Supplement: S2 Fig — A) Indicated peaks refer to calculated mass of respective number of phosphate groups on GST-HAE. Secondary peaks correspond to isoforms with undetermined modifications, presumably from E. coli enzymes. B) Intact mass of MBP-HAE-T851A replicate 1. C) Comparison of total auto-phosphorylation between MBP-HAE-T851A replicate 1 and 2. (TIF) [file pone.0147203.s002.tif]

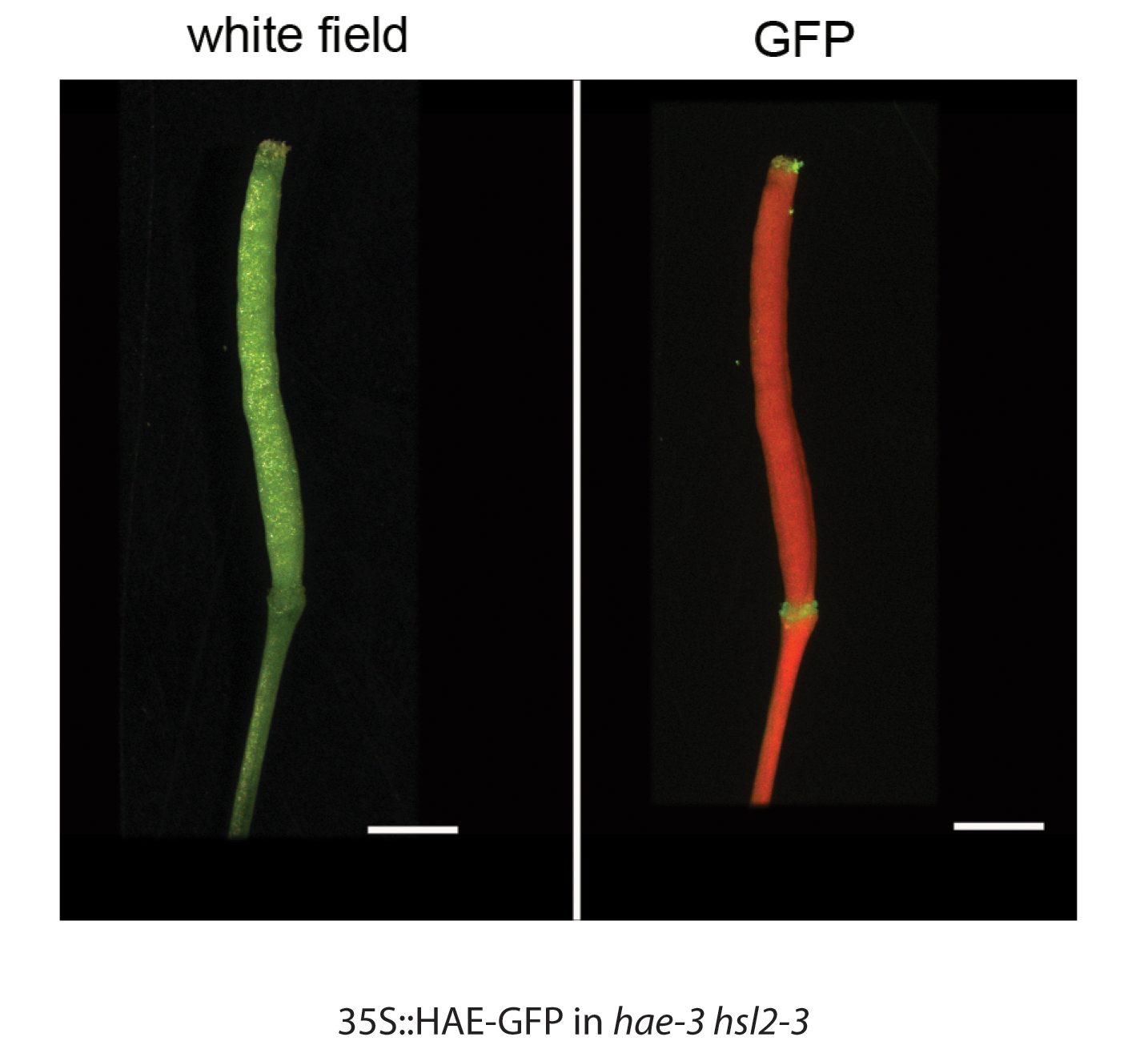

Supplement: S3 Fig — (TIF) [file pone.0147203.s003.tif]

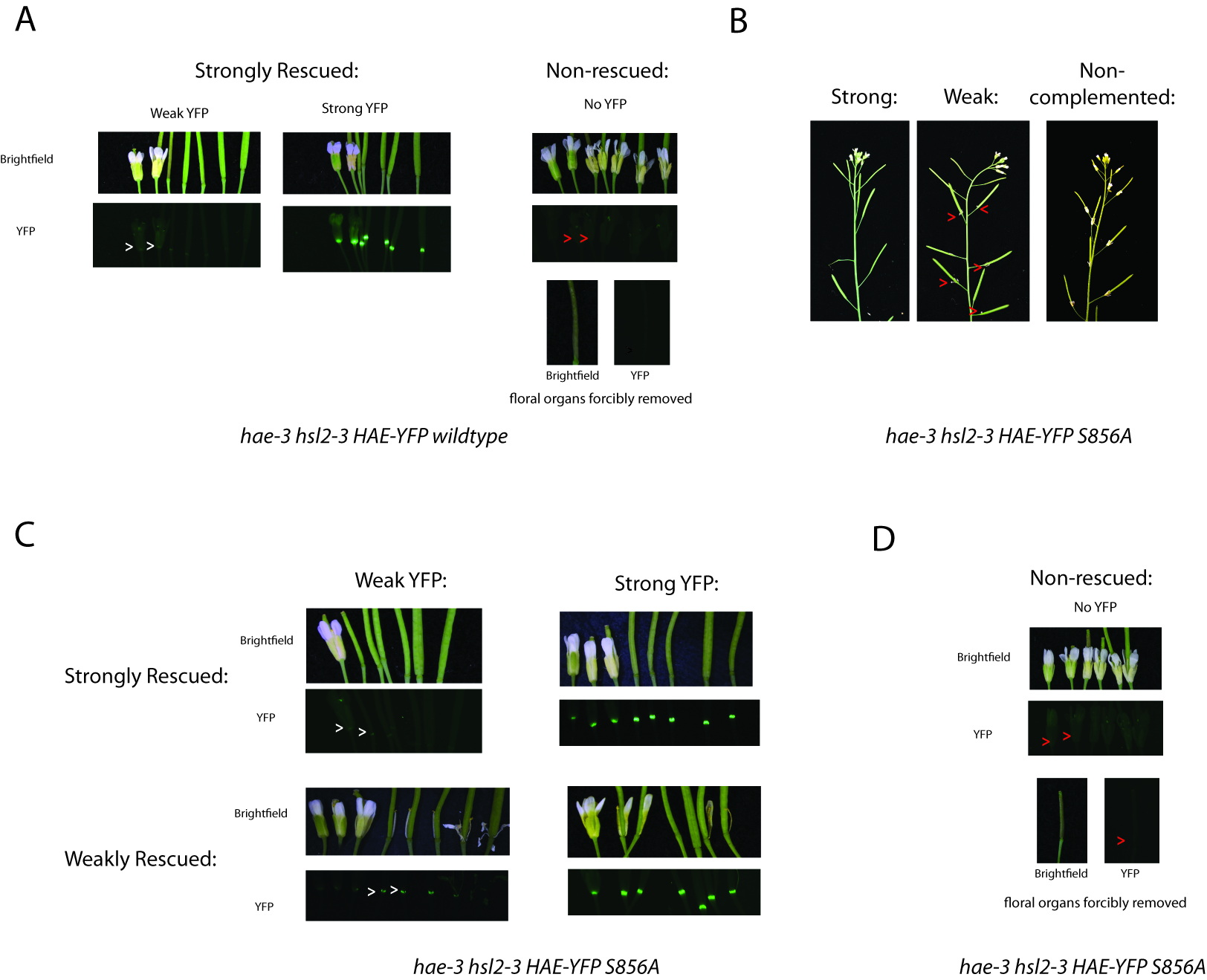

Supplement: S4 Fig — A) Full complementation and non-rescued phenotypes and YFP accumulation of individual T1 plants expressing HAEpr::HAE-YFP wildtype. Arrows point to the abscission zone in fluorescent photographs of weakly expressing and non-expressing lines. B) Representative fully, partially, and non-complemented HAE-YFP S856A T1 lines. Red arrows indicate non-abscised floral organs. C) Abscission phenotype and YFP accumulation for representative fully and partially rescued T1 lines of HAE-YFP S856A. Arrows point to the abscission zone in fluorescent photographs of weakly expressing lines. D) Abscission phenotype and YFP accumulation for representative non-rescued T1 line of HAE-YFP S856A. Arrow points to the abscission zone in fluorescent photograph of a non-expressing line. (TIF) [file pone.0147203.s004.tif]

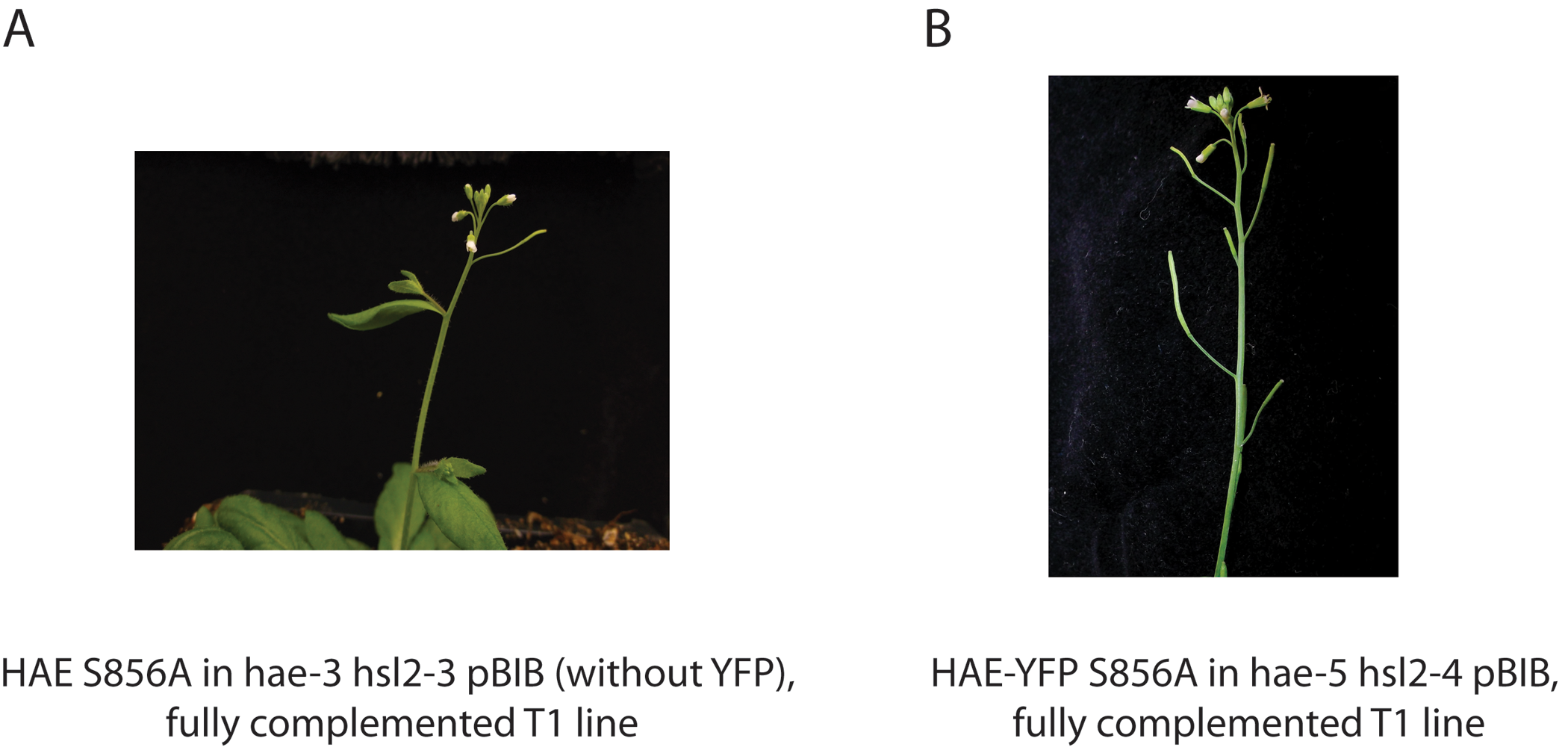

Supplement: S5 Fig — A) Representative plant of HAE-S856A (YFP truncation) in hae-3 hsl2-3. B) Representative T1 plant of HAE-YFP in hae-5 hsl2-4. (TIF) [file pone.0147203.s005.tif]

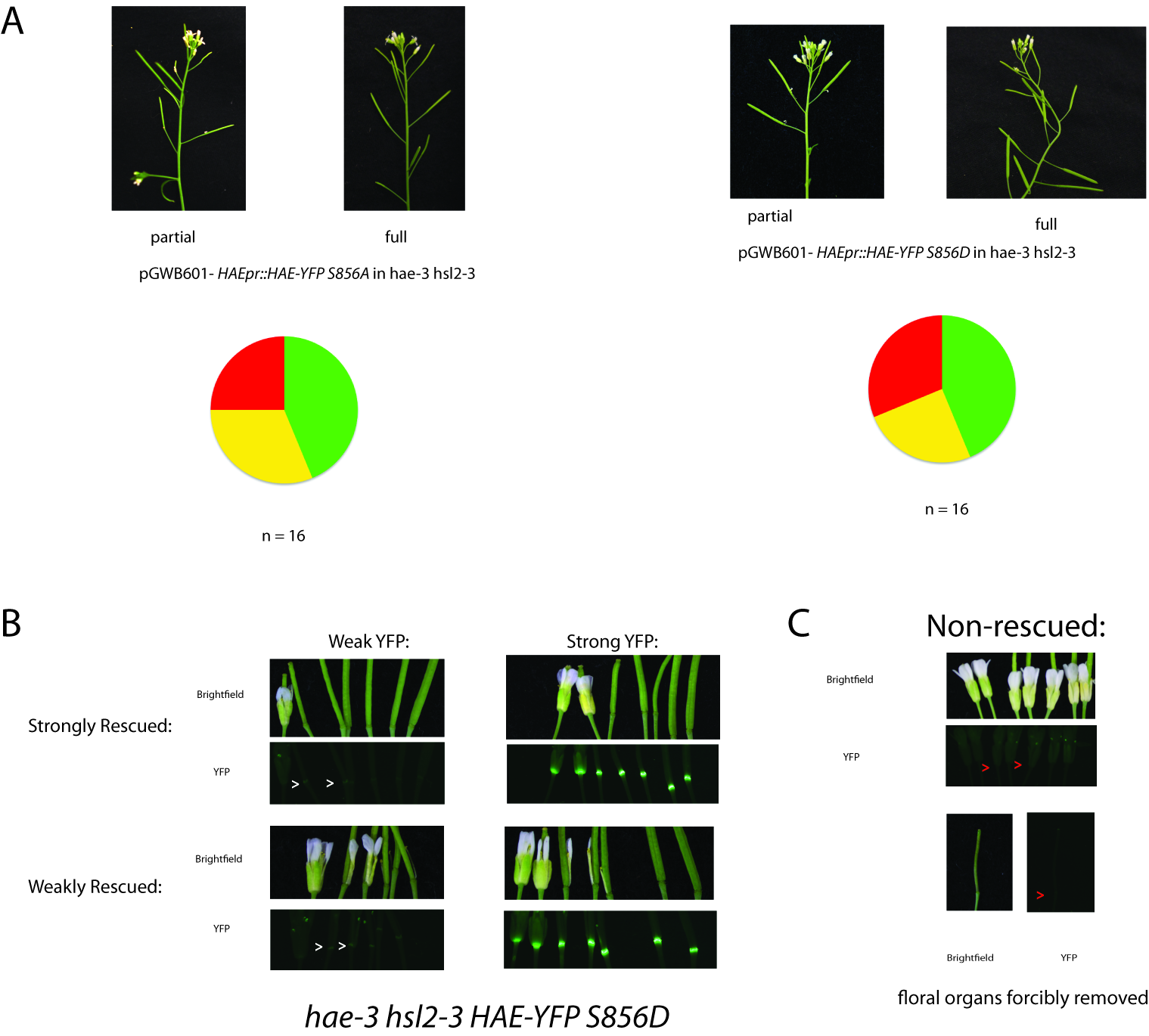

Supplement: S6 Fig — A) Partial and full complementation phenotypes of individual T1 plants expressing HAEpr::HAE-YFP S856A and S856D transgenes. B) Abscission phenotype and YFP accumulation for representative fully and partially rescued T1 lines of HAE-YFP S856D. Arrows point to the abscission zone in fluorescent photographs of weakly expressing lines. C) Abscission phenotype and YFP accumulation for representative non-rescued T1 line of HAE-YFP S856D. Arrow points to the abscission zone in fluorescent photographs of a non-expressing line. (TIF) [file pone.0147203.s006.tif]

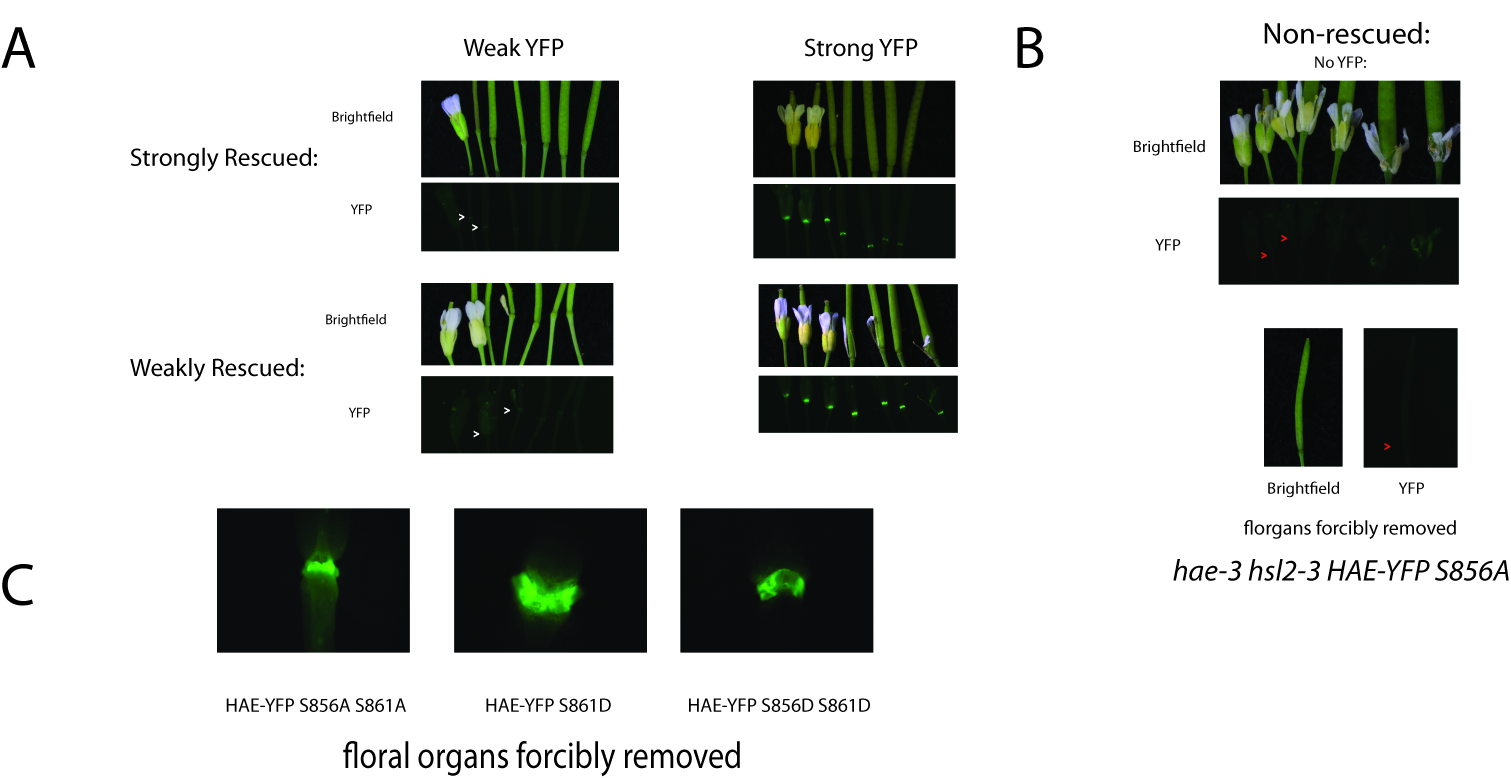

Supplement: S7 Fig — A) Abscission phenotype and YFP accumulation for representative fully and partially rescued T1 lines of HAE-YFP S861A. Arrows point to the abscission zone in fluorescent photographs of weakly expressing lines. B) Abscission phenotype and YFP accumulation for representative non-rescued T1 line of HAE-YFP S861A. Arrow points to the abscission zone in fluorescent photographs of a non-expressing line. C) YFP accumulation in representative T1 lines for the HAE-YFP S856A S861A, HAE-YFP S861D, and HAE-YFP S856D S861D mutants. (TIF) [file pone.0147203.s007.tif]

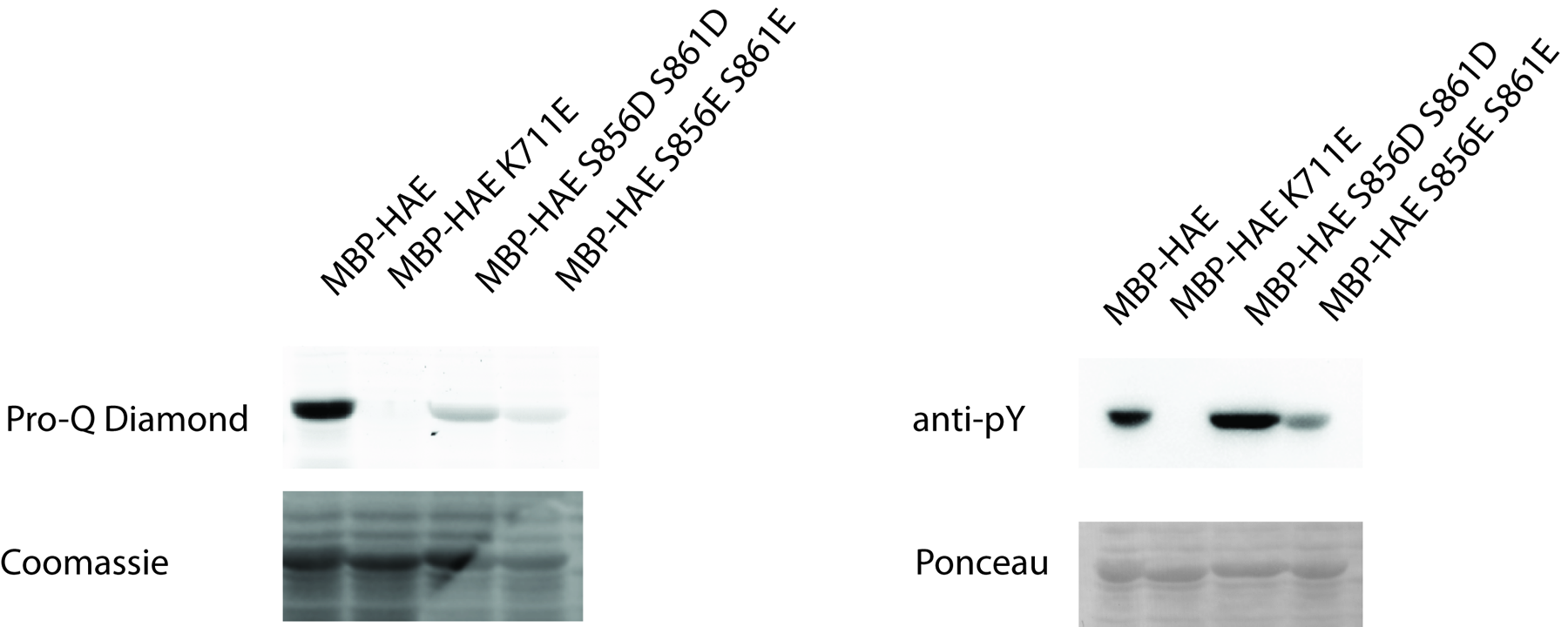

Supplement: S8 Fig — (TIF) [file pone.0147203.s008.tif]

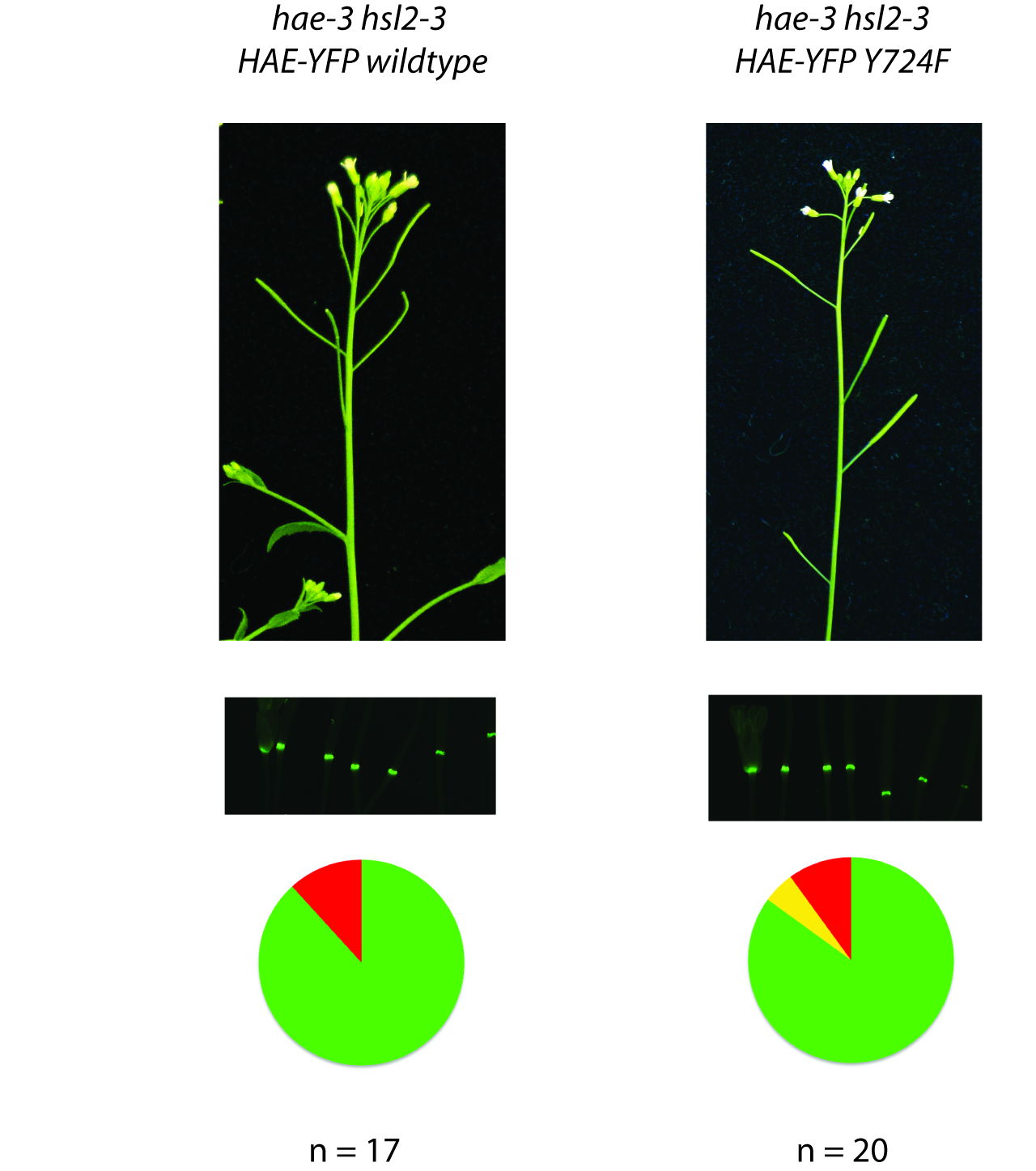

Supplement: S9 Fig — (TIF) [file pone.0147203.s009.tif]

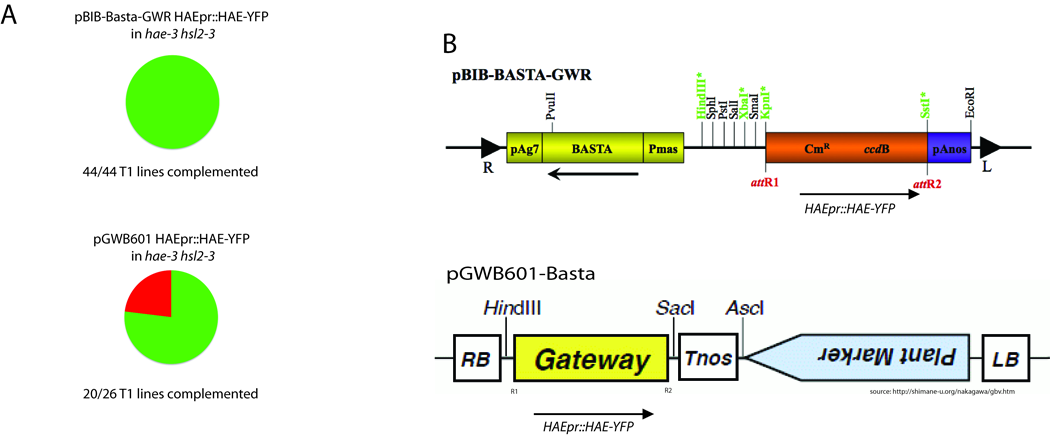

Supplement: S10 Fig — A) A population of pBIB-Basta-GWR HAEpr::HAE-YFP and pGWB601 HAEpr::HAE-YFP T1 transformants was grown side by side to compare complementation efficiency. 44/44 pBIB T1 plants fully abscised, whereas 20/26 pGWB601 T1 plants fully abscised. B) Structure of binary vectors. Arrow labelled “HAEpr::HAE-YFP” indicates direction of recombination from 5’->3’ end of HAE transgene. (TIF) [file pone.0147203.s010.tif]

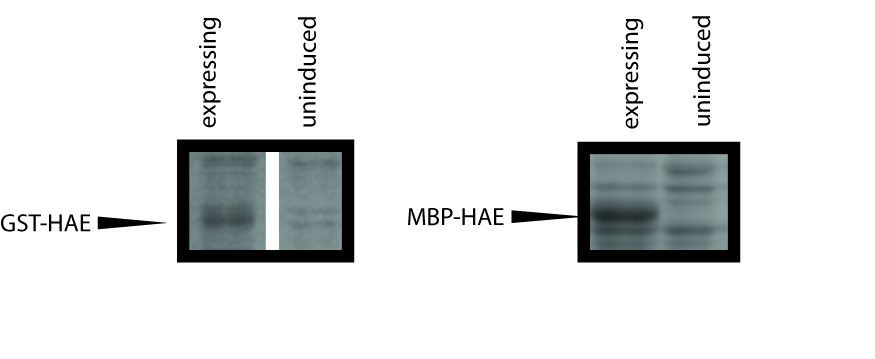

Supplement: S11 Fig — Coomassie stained gels of total cell lysates from GST-HAE and MBP-HAE expressing E. coli. Intervening lanes were deleted from the GST-HAE gel. (TIF) [file pone.0147203.s011.tif]
